# Intermolecular interactions of the malate synthase of *Paracoccidioides spp*

**DOI:** 10.1186/1471-2180-13-107

**Published:** 2013-05-14

**Authors:** Karine Martins de Oliveira, Benedito Rodrigues da Silva Neto, Juliana Alves Parente, Roosevelt Alves da Silva, Guilherme Oliveira Quintino, Aline Raquel Voltan, Maria José Soares Mendes-Giannini, Célia Maria de Almeida Soares, Maristela Pereira

**Affiliations:** 1 Laboratório de Biologia Molecular, Instituto de Ciências Biológicas, Universidade Federal de Goiás, Goiânia, GO, Brazil; 2 Núcleo Colaborativo de BioSistemas, Campus Jatobá, Universidade Federal de Goiás, Goiânia, GO, Brazil; 3 Laboratório de Micologia Clínica, Universidade Estadual Paulista, Araraquara, SP, Brazil

**Keywords:** *Paracoccidioides spp*, Malate synthase, Protein-protein interactions

## Abstract

**Background:**

The fungus *Paracoccidioides spp* is the agent of paracoccidioidomycosis (PCM), a pulmonary mycosis acquired by the inhalation of fungal propagules. *Paracoccidioides* malate synthase (*Pb*MLS) is important in the infectious process of *Paracoccidioides spp* because the transcript is up-regulated during the transition from mycelium to yeast and in yeast cells during phagocytosis by murine macrophages. In addition, *Pb*MLS acts as an adhesin in *Paracoccidioides spp*. The evidence for the multifunctionality of *Pb*MLS indicates that it could interact with other proteins from the fungus and host. The objective of this study was to identify and analyze proteins that possibly bind to *Pb*MLS (*Pb*MLS-interacting proteins) because protein interactions are intrinsic to cell processes, and it might be possible to infer the function of a protein through the identification of its ligands.

**Results:**

The search for interactions was performed using an *in vivo* assay with a two-hybrid library constructed in *S. cerevisiae*; the transcripts were sequenced and identified. In addition, an *in vitro* assay using pull-down GST methodology with different protein extracts (yeast, mycelium, yeast-secreted proteins and macrophage) was performed, and the resulting interactions were identified by mass spectrometry (MS). Some of the protein interactions were confirmed by Far-Western blotting using specific antibodies, and the interaction of *Pb*MLS with macrophages was validated by indirect immunofluorescence and confocal microscopy. *In silico* analysis using molecular modeling, dynamics and docking identified the amino acids that were involved in the interactions between *Pb*MLS and *Pb*MLS-interacting proteins. Finally, the interactions were visualized graphically using Osprey software.

**Conclusion:**

These observations indicate that *Pb*MLS interacts with proteins that are in different functional categories, such as cellular transport, protein biosynthesis, modification and degradation of proteins and signal transduction. These data suggest that *Pb*MLS could play different roles in the fungal cell.

## Background

*In vivo*, the *Paracoccidioides spp* transition from mycelium to yeast cells is governed by an increase in temperature that occurs upon contact of the mycelia or conidia with the host. The fungus, a complex of several phylogenetic species, causes paracoccidioidomycosis (PCM), a human systemic mycosis. The infection begins with the inhalation of fungal propagules, which reach the epithelium of the alveoli, where the mycelium differentiates to the yeast pathogenic form [1]. Although most clinical forms of the disease are asymptomatic, severe and progressive infections involving pulmonary and extra-pulmonary tissues occur [2]. A high percentage (80%) of cases of the disease is reported in Brazil, where PCM is the leading cause of death among the systemic mycoses. PCM is the eighth-leading cause of mortality among infectious and parasitic diseases, which establishes it as a serious public health problem [3-5].

*Paracoccidioides* malate synthase (*Pb*MLS) appears to be important to the infectious process of *Paracoccidioides spp* because the transcript is up-regulated during the transition from mycelium to yeast, during the infectious phase [6], and in yeast cells during phagocytosis by murine macrophages [7]. *Pb*MLS participates in the glyoxylate pathway, which enables the fungus to assimilate two-carbon compounds, and in the allantoin degradation pathway of the purine metabolism, which allows the fungus to use nitrogen compounds [8]. In addition to being a crucial enzyme in the metabolism of *Paracoccidioides spp, Pb*MLS is located in peroxisomes and in the cell wall of the fungus. It is capable of binding to extracellular matrix components such as fibronectin and collagen types I and IV and is also secreted by the fungus. Furthermore, it has been demonstrated that this enzyme plays a role as an adhesin, having the ability to mediate host cell adhesion and internalization of *Paracoccidioides spp* in a significant role in the establishment of infection [9]. Therefore, there is evidence of *Pb*MLS functionality, which drives the investigation of these functions through studies of protein interactions.

The availability of all of the sequences of the *Paracoccidioides spp* genome and the appearance of various techniques for the screening of protein-protein interactions makes it possible to discover the functions of fungal proteins of interest from the identification of their ligands [10]. Therefore, this study was performed to identify *Paracoccidioides spp* proteins that might interact with *Pb*MLS through techniques such as the yeast two-hybrid system (which is the most suitable method for identifying binary interactions) and affinity purifications coupled with mass spectrometry (MS) analyses (pull-down), to discover multi-protein assemblies that enable us to infer other functions of this enzyme and corroborate evidence of their multiple locations in the fungal cell. The interactions were also evaluated by *in silico* analysis.

## Results

### Tracking of protein interactions *in vitro* by pull-down assays

The pull-down technique detects the physical interactions between proteins most directly; as a result, it is a useful tool in the confirmation of protein-protein interactions predicted by other techniques [11]. Here, pull-down assays were performed to search for interactions between *Pb*MLS and other proteins of *Paracoccidioides Pb*01 from different extracts because the fungus expresses different proteins depending on the phase [12], which could lead to different *Pb*MLS-interacting proteins.

The recombinant proteins GST and *Pb*MLS fused to GST (*Pb*MLS-GST) were expressed, purified by using an affinity resin, and visualized by SDS-PAGE (Additional file 1: Figure S1A, lanes 1 and 2, respectively). The predicted mass for the hybrid protein *Pb*MLS-GST was 86.4 kDa (60.9 kDa for *Pb*MLS and 25.5 kDa for GST). The proteins designated as 1, 2, 3 and 4 were subjected to proteolysis and identification by MS. The proteomic identification data are compiled in Additional file 2: Table S1. The results indicated that proteins 1 and 2 correspond to *Pb*MLS (both are PAAG_04542), but protein 2 is most likely a result of its proteolysis or incomplete translation. Protein 3 was identified as membrane protein F of *E. coli*. The co-purification of proteins from *E. coli* has been described [13]. Protein 4 corresponds to GST.

After purification, the GST bound to resin was incubated with protein extracts from *Paracoccidioides Pb*01 mycelium (Additional file 1: Figure S1B), yeast (Additional file 1: Figure S1C), yeast-secreted (Additional file 1: Figure S1D) and macrophage (Additional file 1: Figure S1E), to exclude nonspecific bindings that occur only in the presence of GST. The presence of only GST in lane 1 (Additional file 1: Figures S1B, S1C, S1D and S1E) indicated the absence of non-specific bindings to GST. Next, the supernatant was removed and incubated with *Pb*MLS-GST bound to resin. The protein complexes formed during incubation were precipitated and resolved by SDS-PAGE (lane 2 – Additional file 1: Figures S1B, S1C, S1D and S1E).

Proteins that interacted with *Pb*MLS, which are listed from 5 to 66 (Additional file 1: Figure S1B, S1C, S1D and S1E), were removed from the gel and identified by MS (Additional file 2: Table S1). Proteins that interact with *Pb*MLS and that were detected by different pull-down assays were listed (Additional file 3: Table S2). The search against the NCBI non-redundant database using the MS/MS data was performed using MASCOT software v. 2.4 [14]. Functional characterization was performed using UniProt databases [15] and MIPS [16].

A total of 45 *Pb*MLS-interacting proteins were identified (Additional file 3: Table S2). Of these, 18 proteins were from macrophage and 27 were from *Paracoccidioides Pb*01; 15 were from mycelium, 18 were from yeast, and 11 were yeast-secreted. Some proteins were found in more than one extract (4 proteins in mycelium, yeast and yeast-secreted, 11 proteins in mycelium and yeast, 1 protein in mycelium and yeast-secreted). No protein was found in both yeast and yeast-secreted extracts. Of the 27 *Paracoccidioides Pb*01 proteins, 13 were exclusively extract (found only in mycelium, yeast or yeast-secreted). Of 18 macrophage proteins, 13 were exclusive to macrophage, with 5 related to cytoskeleton. A total of 3 proteins (heat shock protein 60 kDa, heat shock protein 70 kDa and fructose 1, 6 bisphosphate aldolase) were also identified in the pull-down assays with *Paracoccidioides Pb*01 mycelium and/or yeast cells.

### Tracking of protein interactions *in vivo* by a two-hybrid assay

To detect new interactions between *Pb*MLS and other *Paracoccidioides Pb*01 proteins, two-hybrid assays were performed. The Y187 strain of *S. cerevisiae* that harbors the bait (*Pb*MLS) fused to the binding domain (BD) of the GAL4 transcription factor and the strain AH109 that harbors the prey (cDNA library of *Paracoccidioides Pb*01) fused to the activation domain (AD) of GAL4 were placed in the same system to promote diploids.

The diploid yeast-expressing proteins that interacted were finally selected in medium that contained a chromogenic substrate (X-α-GAL) to observe the transcriptional activation of the reporter gene *mel1*, a GAL4-regulated gene coding for the α-galactosidase enzyme. A total of 24 clones showed the activation of the reporter gene *mel1* by turning blue (data not shown), which confirmed that there was interaction between *Pb*MLS and the gene products listed in the Additional file 4: Table S3.

To identify gene products that interacted with *Pb*MLS, the cDNAs of the clones were sequenced after PCR amplification. ESTs (Expressed Sequence Tags) were processed using the bioinformatics tool Blast2GO. The functional classification was based on the homology of each EST against the GenBank database using the BLAST algorithm [17], with a significant homology cutoff of ≤ 1e^-5^ and functional annotation by MIPS [16]. Additionally, sequences were grouped into functional categories through the PEDANT 3 database [18]. The analysis indicated the presence of several functional categories of genes and cell functions related to cellular transport, protein fate, protein synthesis, nucleotide metabolism, signal transduction, cell cycle and DNA processing, and hypothetical protein (Additional file 4: Table S3).

### Construction of protein interaction maps

**Figure 1:**
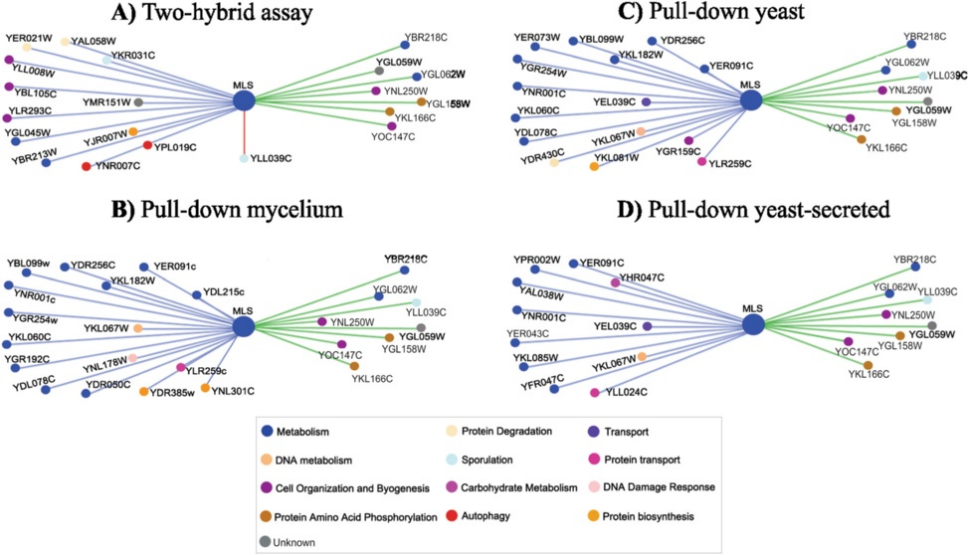
**Map of interactions between MLS and other proteins generated by the Osprey Network Visualization System **[25]**.** (**A**) Protein interactions obtained by a two-hybrid assay. Protein interactions obtained by pull-down assays with protein extracts of *Paracoccidioides* mycelium (**B**), yeast (**C**) and yeast secretions (**D**). The blue lines indicate protein interactions with MLS from the experimental data. The green lines indicate protein interactions with MLS that are already described in The GRID interaction database [24] of *S. cerevisiae*. The pink line corresponds to both. The colored dots show the functional classifications of the proteins.

A comprehensive genetic interaction dataset has been described for the model yeast *S. cerevisiae*[19]. Because genes that act in the same pathway display similar patterns of genetic interactions with other genes [19-22], we investigated whether *Paracoccidioides Pb*01 protein sequences that interacted with *Pb*MLS and were tracked by the pull-down and two-hybrid assays (Additional file 3: Table S2 and Additional file 4: Table S3, respectively) were found in the structural genome database of *S. cerevisiae*[23]. Those sequences and others from The GRID protein interaction database [24] of *S. cerevisiae* were used to construct protein interaction maps generated by the Osprey Network Visualization System [25] (Figure 1). Protein sequences from macrophage were not used because some of them were not found in the *S. cerevisiae* database. The blue lines indicate protein interactions with MLS from *Paracoccidioides Pb*01 experimental data. The green lines indicate protein interactions with MLS already described in The GRID interaction database [24] of *S. cerevisiae*. A pink line corresponds to both. The colored dots show the functional classification of proteins.

Protein interactions obtained by a two-hybrid assay are shown in Figure 1A. Protein interactions obtained by pull-down assays with protein extracts of *Paracoccidioides Pb*01 mycelium, yeast and yeast-secretions are shown in Figure 1B, C, and D, respectively. Ubiquitin (YLL039C) was the only protein that interacted with MLS that was found in both *Paracoccidioides* and *S. cerevisiae*. The other proteins were identified in *Paracoccidioides Pb*01 or *S. cerevisiae* but not in both. Although some proteins identified in *Paracoccidioides Pb*01 have homologous proteins in *S. cerevisiae* (Additional file 5: Table S4), these proteins could not yet be identified as interacting with *Pb*MLS. Most of the *Paracoccidioides Pb*01 proteins that interacted with *Pb*MLS were related to the metabolism category.

### Confirmation of the interactions by Far-Western blot assays

**Figure 2:**
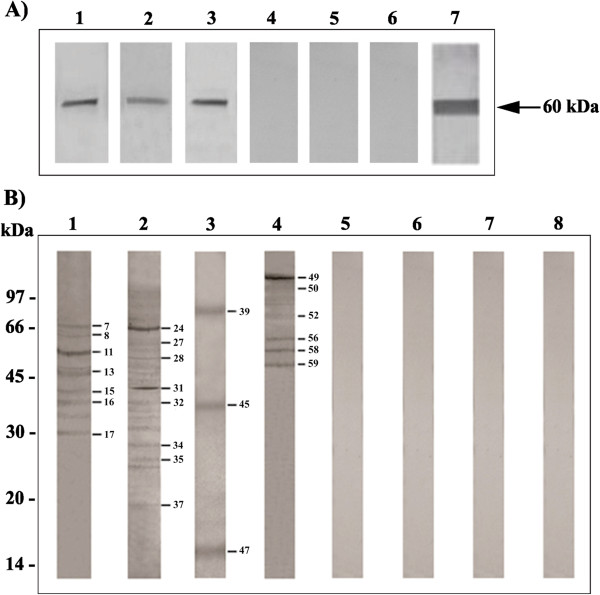
**Confirmation of the interactions by Far-Western blot assays.** (**A**) *Pb*MLS was subjected to SDS-PAGE and electro blotted. Membranes were reacted with *Paracoccidioides* protein extracts of mycelium (lane 1), yeast (lane 2) and macrophage (lane 3) and were subsequently incubated with anti-rabbit IgG anti-enolase, anti-triosephosphate isomerase and anti-actin, respectively. The reactions were revealed with anti-rabbit IgG conjugated to alkaline phosphatase. Negative control was obtained by incubating *Pb*MLS with the antibodies anti-enolase, anti-triosephosphate isomerase and anti-actin, respectively, without preincubation with the protein extracts (lanes 4, 5 and 6). The positive control was obtained by incubating the *Pb*MLS with the polyclonal anti-*Pb*MLS antibody (lane 7). (**B**) Protein extracts of *Paracoccidioides* mycelium, yeast, secretions and macrophages (lanes 1, 2, 3 and 4, respectively) were subjected to SDS-PAGE and blotted onto nylon membrane. The membranes were incubated with *Pb*MLS and, subsequently, primary antibody anti-*Pb*MLS and secondary antibody anti-rabbit IgG. Negative control was obtained by incubating each protein extract with anti-*Pb*MLS antibody, without preincubation with *Pb*MLS (lanes 5, 6, 7 and 8). The numbers indicate the proteins (Additional file 2: Table S1) that interact with *Pb*MLS that are confirmed by this technique.

Far-Western blot assays were conducted to confirm the interactions between *Pb*MLS and other proteins from the fungus identified by pull-down assays. *Pb*MLS was subjected to SDS-PAGE and was electro blotted. The membranes were reacted with protein extracts of *Paracoccidioides Pb*01 mycelium, yeast and macrophage (Figure 2A, lanes 1, 2 and 3, respectively) and were subsequently incubated with rabbit IgG anti-enolase, anti-triosephosphate isomerase and anti-actin, respectively. The reactions were revealed with anti-rabbit IgG conjugated to alkaline phosphatase. Positive signals to the three extracts indicated the presence of an interaction between *Pb*MLS and enolase, triosephosphate isomerase and actin. Negative control was obtained by incubating *Pb*MLS with the antibodies anti-enolase, anti-triosephosphate isomerase and anti-actin, respectively, without preincubation with the protein extracts (Figure 2A, lanes 4, 5 and 6, respectively). Positive control was obtained by incubating the *Pb*MLS with the polyclonal anti-*Pb*MLS antibody (Figure 2A, lane 7).

Another Far-Western blot assay was performed using membranes that contained protein extracts of *Paracoccidioides Pb*01 mycelium, yeast, yeast secretions, and macrophage (Figure 2B, lanes 1, 2, 3 and 4, respectively). The membranes were incubated with *Pb*MLS and, subsequently, were incubated with antibody anti-*Pb*MLS and secondary antibody anti-rabbit IgG. Several proteins identified in the pull-down assays interacted with *Pb*MLS at this point, which suggested the veracity of the interactions. Negative control was obtained by incubating each protein extract with the anti-*Pb*MLS antibody, without preincubation with *Pb*MLS (Figure 2B, lanes 5, 6, 7 and 8). The numbers identify the proteins that interacted with *Pb*MLS, as shown in Additional file 2: Table S1.

### *Pb*MLS binds to the surface of macrophages

**Figure 3:**
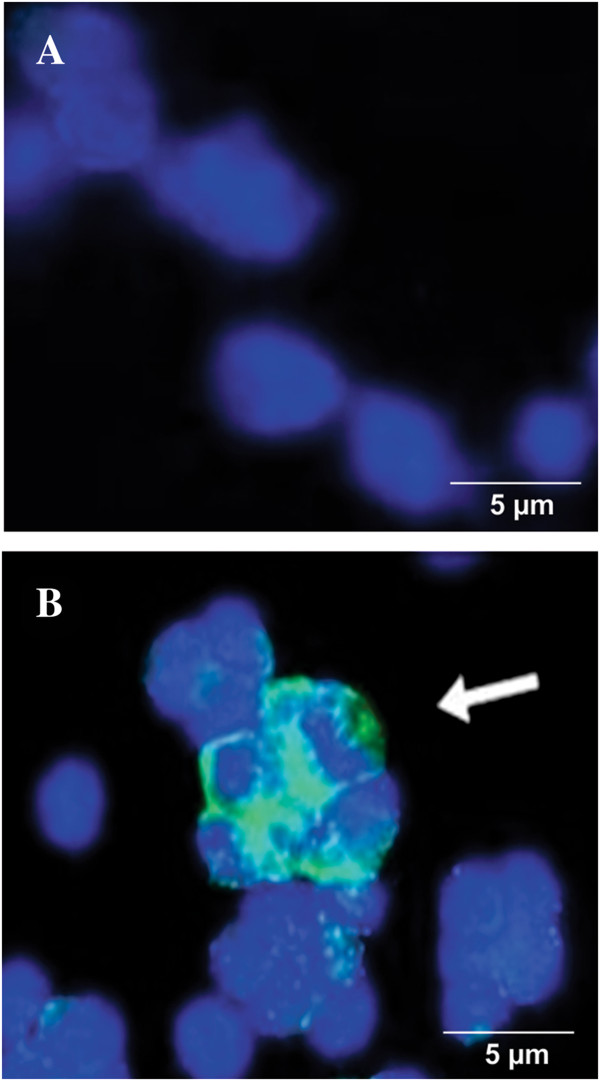
**Binding of *Pb*MLS to the macrophage surface.** Immunofluorescence microscopy that shows the binding of *Pb*MLS to J774 A.1 mouse macrophage cells. (**A**) Negative control was performed with the unrelated protein BSA. (**B**) Arrows indicate *Pb*MLS (green) binding to the macrophage cell surfaces; blue indicates the macrophage nucleus.

Because the results from Far-Western blot assays revealed several macrophage proteins interacting with *Pb*MLS, we performed immunofluorescence microscopy to visualize whether *Pb*MLS could adhere to the surface of the macrophage cells. No binding was observed using BSA as a control (Figure 3A). The arrow indicates *Pb*MLS binding to a macrophage surface (Figure 3B).

### *Pb*MLS participates in the adherence of *Paracoccidioides* to pneumocyte cells

**Figure 4:**
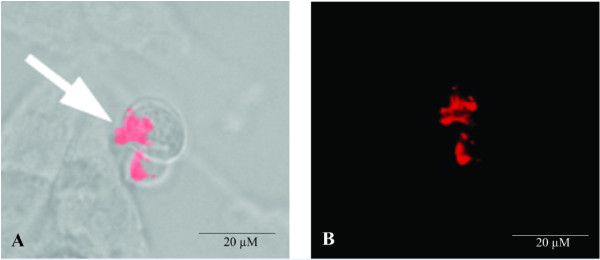
**Interaction between *Paracoccidioides *yeast cells and pneumocytes by confocal laser scanning microscopy.** Infected cell monolayers were fixed and permeabilized. Primary anti-*Pb*MLS and secondary antibodies Alexa Fluor 594 goat anti-rabbit IgG (red) were used. The specimens were analyzed by laser confocal microscopy using DIC (**A**) and fluorescence (**B**).

Because the fungus initially reaches the lungs, the participation of *Pb*MLS in the adherence of *Paracoccidioides Pb*18 to pneumocyte cells was investigated by using confocal laser scanning microscopy. A549 cells were pretreated with anti-*Pb*MLS and infected with *Paracoccidioides Pb*18 isolate. After washings with frozen PBS-T, the monolayers were incubated with Alexa Fluor that was 594-conjugated for labeling the antibody. The arrows indicate *Pb*MLS interacting with the A549 surface (Figures 4A and B).

### Homology models

*In silico* analysis was performed to investigate how the interactions identified by pull-down and two-hybrid assays could occur. Some *Pb*MLS-interacting proteins from metabolic pathways such as the glycolytic pathway, the tricarboxylic acid cycle, the methyl citrate cycle and the glyoxylate cycle were selected for analysis. Because *Pb*MLS participates in the glyoxylate cycle, interaction between proteins from different metabolic pathways would be expected. Because no crystal structure of *Pb*MLS-interacting proteins described here was reported, a three-dimensional homology model for each protein was constructed based on the structure template listed in Additional file 6: Table S5. All of the 3D-structure templates used to build models of the proteins have a resolution of < 2.0 Å and an identity of > 49%, with a coverage of > 91%.

Homology models of the *Pb*MLS-interacting proteins have very little conformational change when compared to their templates (Additional file 6: Table S5). The largest deviations were observed for enolase and fructose 1,6 bisphosphate aldolase, with 2.65 Å and 1.44 Å of root mean square derivation (RMSD) when superposed on the template when considering the non-hydrogen atoms. For enolase, there is a significant conformational change only in the C-terminal regions and between PRO143 and ASN155 (data not shown).

Alpha-helix-like secondary-structure patterns were observed in a greater proportion in the homology models *Pb*MLS-interacting proteins. For almost all of the structures, the alpha-helix-like pattern corresponded to more than 40% of the whole structure, while the beta-sheet-like pattern accounted for less than 20%, except for the protein ubiquitin, whose quantity of beta-sheet-like pattern was greater (Additional file 6: Table S5).

Ramachandran plots of homology models were assessed stereo-chemically through the RAMPAGE web server [26] (data not shown). For all of the proteins, the Φ and Ψ distributions of the Ramachandran plots were always above 94% in the favored regions and less than 3.5% in the allowed regions. The quality factors of the structures were estimated by the ERRAT web server and are summarized in Additional file 6: Table S5.

### Molecular dynamics

All of the proteins were subjected to at least 20 ns simulation using GROMACS software [27]. For the proteins gamma actin, 2-methylcitrate synthase, triosephosphate isomerase and ubiquitin, that time was insufficient to achieve RMSD stability of non-hydrogen atoms with respect to the structure homology models. In those cases, more simulation time was provided until this condition was achieved. The times required are listed for each protein. For almost all of the proteins, the deviations from their homology models were low (approximately 3.0 Å). Specifically, ubiquitin and 2-methylcitrate synthase had the highest RMSDs. The increase was 7.65 Å and 6.34 Å after 60 ns and 40 ns, respectively. When only the residues from the interfaces of the complexes were considered, the RMSDs increased 9.0 Å and 5.87 Å, respectively (Additional file 6: Table S5).

The alpha-helix-like pattern was slightly reduced in all of the proteins that were binding to *Pb*MLS, but the beta-sheet-like structures almost did not change. Although the RMSDs were high for ubiquitin and 2-methylcitrate synthase, the alpha-helix-like patterns decreased to only 10.6% and 6.9%, respectively.

### Molecular docking and molecular dynamics of the protein-protein complexes

**Figure 5:**
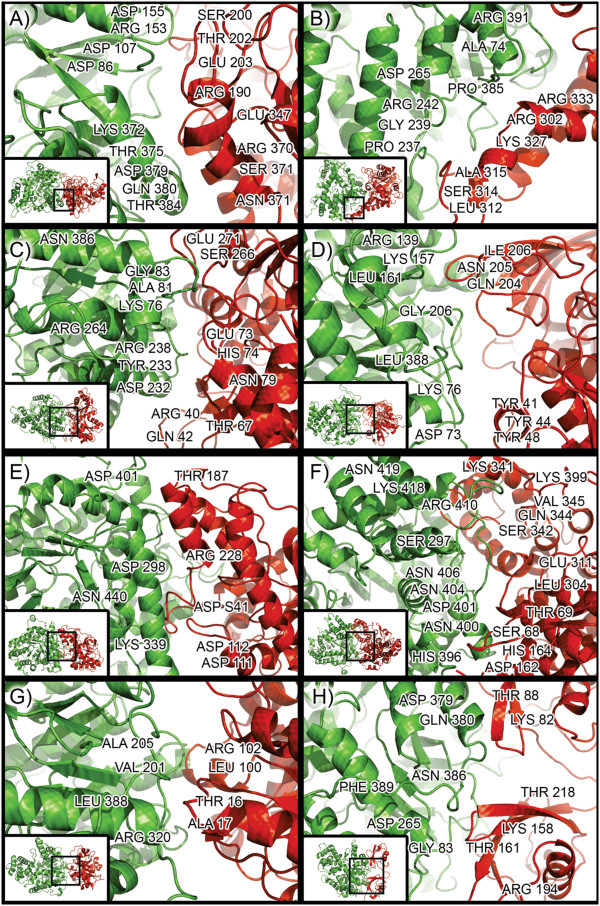
**Complexes between *Pb*MLS-interacting proteins (red) and *Pb*MLS (green) after protein-protein docking simulations by using Gramm-X and GROMACS software.** (**A**) Enolase, (**B**) Fructose 1, 6 bisphosphate aldolase, (**C**) Gamma actin, (**D**) Glyceraldehyde-3-phosphate isomerase, (**E**) Malate dehydrogenase, (**F**) 2-Methylcitrate dehydratase, (**G**) Triosephosphate isomerase, and (**H**) Ubiquitin. The amino acid residues that are involved in complex formation are highlighted.

Molecular docking between *Pb*MLS and *Pb*MLS-interacting proteins was investigated by the GRAMM-X web server using the structures stabilized by DM. Only the best model-structures provided by the server were selected. These complexes were then subjected to a rapid DM so that their structures could accommodate and avoid high energy at the interface between them, thus identifying residues in this region. Significant conformational changes occurred in ubiquitin and 2-methylcitrate synthase when they were complexed with *Pb*MLS (data not shown). The residues contacting at the interface of the complexes are shown in Additional file 7: Table S6, and these amino acids are highlighted in Figure 5. Some amino acid residues are common to different proteins. For example, ASP379 and GLN380 are residues of *Pb*MLS that interact with enolase and ubiquitin; ASN386 is at the interface for gamma actin and ubiquitin; LEU388 is common to triosephosphate isomerase and glyceraldehyde-3-phosphate dehydrogenase; and ASP401 is common to 2-methylcitrate synthase and malate dehydrogenase.

The protein-protein complexes relaxed by DM were provided to the Fiberdock web server, which determined the global energy for each complex (Additional file 7: Table S6). The results showed that fructose 1, 6 bisphosphate aldolase and ubiquitin were well stabilized when complexed with *Pb*MLS. The ASP265 residue of *Pb*MLS is present in the interaction of both proteins.

## Discussion

Our previous studies showed that *Pb*MLS is required in the metabolism of *Paracoccidioides Pb*01 acting in the glyoxylate cycle and in the allantoin degradation pathway. *Pb*MLS condenses acetyl-CoA from both 2C sources (glyoxylate cycle) and nitrogen sources (from proline and purine metabolism) to produce malate, which is a central molecule of the tricarboxylic acid cycle or glyoxylate cycle [8]. In addition, *Pb*MLS is located in the cytoplasm and on the fungal cell surface and is secreted, behaving like an anchorless adhesin [9]. The strong evidence for *Pb*MLS multifunctionality increased our interest in researching the possibility of new roles for *Pb*MLS through studies of protein-protein interactions, which aimed to identify *Pb*MLS-interacting proteins.

We searched for *Pb*MLS-interacting proteins using Far-Western blot, pull-down and two-hybrid techniques. The two-hybrid and pull-down are used as complementary techniques because the results depend on variants of the methods. The two-hybrid system is highly sensitive to detecting low-abundance proteins, unlike the pull-down system, which detects high-abundance molecules. Additionally, the two-hybrid system allows identifying strong and weak interactions, while the pull-down is not a sensitive method for identifying some of the weak interactions because of the wash steps [28]. Because the principles of the techniques are different, we have the capability of identifying different proteins.

Pull-down assays were performed using *Paracoccidioides Pb*01 mycelium, yeast and yeast-secreted protein extracts because protein differences [12] and metabolic differences, including changes in the *Pb*MLS transcript expression level [29], were observed between both phases, which could lead to different *Pb*MLS-interacting proteins. In fact, considering mycelium and yeast, 4 proteins were exclusive to mycelium, and 7 were exclusive to yeast. In addition, 5 proteins were exclusive to yeast-secreted extract, and 15 were exclusive to macrophage. A total of 13 of those proteins were also identified by Far-Western blot. These findings suggest that *Pb*MLS appears to play a different role in *Paracoccidioides Pb*01 because it interacts with proteins from diverse functional categories.

Several significant interactions were found. *Pb*MLS interacted with fatty acid synthase subunit beta, which catalyzes the synthesis of long-chain saturated fatty acids. *Pb*MLS interacted with 2-methylcitrate synthase and 2-methylcitrate dehydratase, which are enzymes of the cycle of 2-methylcitrate. This cycle is related to the metabolism of propionyl-coenzyme A (and odd-chain fatty acids), unlike the glyoxylate cycle, which is related to the metabolism of even-chain fatty acids. The interaction of *Pb*MLS with these enzymes suggests its involvement in fatty acid metabolism regulation.

The peroxisomal enzyme malate dehydrogenase, which participates in the glyoxylate cycle [30], interacts with *Pb*MLS. In addition to having the signal peptide AKL that targets peroxisomes [8], *Pb*MLS was localized in that organelle [9].

*Pb*MLS interacts with serine threonine kinase. It is known that protein kinases catalyze the transfer of the gamma phosphate of nucleotide triphosphates (ATP) to one or more amino acids of the protein side chain, which results in a conformational change that affects the function of the protein, resulting in a functional alteration of the target protein by altering enzymatic activity, cellular localization or association with other proteins [31]. Thus, the interaction with a protein kinase suggests that *Pb*MLS could be regulated by phosphorylation. *Pb*MLS has a variety of sites, which indicates possible post-translational modifications, including protein kinase phosphorylation sites [8]. We have already described the regulation by phosphorylation of *Pb*ICL, the other enzyme unique to the glyoxylate cycle [32].

The secretion of *Pb*MLS [9] suggests that it interacts with fungus proteins themselves and host surface proteins. Extracellular vesicles from *Paracoccidioides spp* present proteins with many functions [33]. Of 11 *Pb*MLS-interacting proteins, 5 were also found in the extracellular vesicle. Extracellular proteins are known to play important roles, such as the uptake of nutrients, cell-cell communication and detoxification of the environment [34]. More specifically, proteins secreted by pathogenic microorganisms appear to play important roles in virulence [35]. Corroborating our results, many proteins identified in this study, such as 2-methylcitrate synthase, malate dehydrogenase, nucleoside diphosphate kinase, pyruvate kinase, hsp70-like protein and Cobalamin-independent methionine synthase, had previously been described as secreted proteins in *Paracoccidioides Pb*01 secretome from mycelium and yeast cells [36].

The adhesion of pathogens to host cells is considered to be an essential step in the establishment of infection [37]. Several clinically important fungi, such as *Candida albicans*, *Aspergillus fumigatus*, *Histoplasma capsulatum* and *Cryptococcus neoformans,* are known to bind to proteins of the extracellular matrix (ECM) [38]. The adhesins of fungi are important in the migration, invasion, differentiation and proliferation of microbes. *Paracoccidioides* yeast cells also have the ability to adhere and invade host cells [39,40]. Some adhesins, such as *Pb*Dfg5p [41], triosephosphate isomerase (*Pb*TPI) [42], glyceraldehyde-3-phosphate dehydrogenase (*Pb*GAPDH) [39], and enolase (*Pb*Eno) [43], and *Pb*MLS [9] have been described in *Paracoccidioides Pb*01. Here, the interaction between *Pb*MLS and enolase and triosephosphate isomerase was confirmed by Far-Western blot assay. The interaction of *Pb*MLS with those proteins suggests that the joint action of those adhesins could promote adhesion to and invasion of host cells, acting as potent virulence factors.

*Pb*MLS appears to act in the interaction between *Paracoccidioides Pb*01 and macrophage because it interacts with several macrophage-specific proteins, of which 5 proteins are related to cytoskeleton, which suggests the involvement of that structure in the fungus adhesion process. The *Pb*MLS binding to actin was confirmed by Far-Western blot. The cytoskeletons of the macrophages control the movement of the cell membrane, which reflects the movement of the cell as a whole and are also involved in processes such as phagocytosis [44]. Our previous work used Far-Western blotting and flow cytometry to show that *Pb*MLS binds to A549 cells. Here, the participation of *Pb*MLS in *Paracoccidioides Pb*01 adhesion to and invasion of A549 cells was confirmed using confocal laser scanning microscopy.

Some *Pb*MLS-interacting proteins were selected for *in silico* interaction analysis. Proteins were chosen from metabolic pathways such as the glycolytic pathway, the tricarboxylic acid cycle, the methyl citrate cycle and the glyoxylate cycle because *Pb*MLS participates in the glyoxylate cycle, and the interaction between proteins from different metabolic pathways would be expected. Global energy values for each complex studied showed that there is good complementarity between *Pb*MLS and most *Pb*MLS-interacting proteins. For example, the complexes that involve *Pb*MLS and the proteins glyceraldehyde-3-phosphate isomerase, malate dehydrogenase, 2-methylcitrate dehydratase and triosephosphate isomerase have global energies that are less than −55 kcal/mol. The global energy values found here were very good. For example, in a recent study of the interactions between D-phosphoglycerate dehydrogenase and phosphoserine aminotransferase from the enteric human parasite *Entamoeba histolytica*[45], the best global energies were approximately −75 kcal/mol. Here, the best values were found for fructose 1,6 bisphosphate aldolase and ubiquitin (less than −100 kcal/mol).

*S. cerevisiae* MLS-interacting proteins have already been described. Here, *in silico* analysis using the *S. cerevisiae* database showed that *Pb*MLS interacts with other new proteins. The only protein that they share is ubiquitin. This fact and the fact that the interaction between ubiquitin and *Pb*MLS is very stable suggest that this interaction is very important. Ubiquitin is responsible for the conjugation of proteins, marking them for selective degradation via the ubiquitin-proteasome system 26S, a process that is essential in the response to cellular stress. These proteins, however, act through ubiquitination, changing the function, the location and/or the traffic protein, or are targeted for destruction by the 26S proteasome [46].

In conclusion, the molecular interactions that involve proteins located in subcellular compartments facilitate the understanding of mechanisms that are associated with each interaction. However, proteins are not always at the same location in the cell and do not have unique roles [47]. Here, several new *Pb*MLS-interacting proteins from various functional categories were identified, which suggests that their function is diversified beyond the glyoxylate cycle.

## Conclusions

The results of this study indicated that *Pb*MLS interacts with proteins of different functional categories, such as cellular transport, protein biosynthesis, modification and degradation and signal transduction. These data suggest that *Pb*MLS is found in many locations and plays different roles in the fungal cell.

## Methods

### *Paracoccidioides* isolate and growth conditions

The fungus *Paracoccidioides* isolate *Pb*01 (ATCC MYA-826) was grown, as previously described [39]. The yeast and mycelium phase were grown at 36 and 22 °C, respectively, in Fava–Netto’s medium (1% w/v peptone, 0.5% w/v yeast extract, 0.3% w/v proteose peptone, 0.5% w/v beef extract, 0.5% w/v NaCl, 4% w/v glucose, 1% w/v agar pH 7.2).

### Preparation of protein extracts from *Paracoccidioides spp*

Total protein extracts from *Paracoccidioides spp* mycelium and yeast cells were prepared as previously described [48]. Mycelium and yeast cells were frozen and ground with a mortar and pestle in buffer (20 mM Tris–HCl pH 8.8, 2 mM CaCl_2_) with protease inhibitors (50 μg/mLN-α-ρ-tosyl-_L_-lysine chloromethylketone; 1 mM 4-chloromercuribenzoic acid; 20 mM leupeptin; 20 mM phenylmethylsulfonyl fluoride; and 5 mM iodoacetamide). The mixture was centrifuged at 10,000 × *g* at 4°C, for 20 min, and the supernatant was collected and stored at −20 °C.

Yeast-secreted proteins of *Paracoccidioides spp* were prepared. Culture supernatant of yeast cells was obtained after 24 h incubation in liquid Fava Netto’s medium. The cells were separated by centrifugation at 5,000 × *g* for 15 min, and the supernatant was filtered in 0.45 and 0.22 μm filters (MilliPore). Each 50 mL of culture supernatant was concentrated to 500 μL in 25 mM Tris–HCl pH 7.0, and a protease inhibitor was added. The protein concentration of all of the samples was determined according to Bradford [49].

### Preparation of protein extracts from macrophage

J774 A.1 mouse macrophage cells purchased from a Cell Bank in Rio de Janeiro, Brazil [50], were cultured in RPMI 1640 supplemented with fetal bovine serum, nonessential amino acids and interferon gamma (1 U/mL). To obtain the protein extract, cells were detached with 0.9% saline solution containing trypsin and were centrifuged at 5,000 × *g* for 10 min. Then, milliQ water was added to lyse the cells, and the solution was centrifuged again. Buffer (20 mM Tris–HCl pH 8.8, 2 mM CaCl_2_) and protease inhibitors were added to the pellet. Protein concentration was determined according to Bradford [49].

### Heterologous expression and purification of recombinant *Pb*MLS

*Pb*MLS recombinant protein was obtained as described by Zambuzzi-Carvalho *et al.*[8] and Neto *et al*. [9]. *Pb*MLS cDNA was cloned into the expression vector pGEX-4-T3 (GE Healthcare®, Chalfont St Giles, UK). *E. coli* (BL21 Star™ (DE3) pLys, Invitrogen, Grand Island, NY) was transformed with pGEX-*Pb*MLS construction by thermal shock and was grown in LB medium supplemented with ampicillin (100 μg/mL) at 20°C until reaching the optical density of 0.6 at 600 nm. Synthesis of the recombinant protein was then initiated by adding isopropyl-β-D-thiogalactopyranoside (IPTG) (Sigma-Aldrich, St. Louis, MO) to a final concentration of 0.1 mM to the growing culture. After induction, the cells were incubated for 16 h at 15°C with shaking at 200 rpm. Cells were harvested by centrifugation at 10,000 × *g* for 10 min. The supernatant was discarded, and the cells were resuspended in 1× phosphate-buffered saline (PBS) (0.14 M NaCl, 2.7 mM KCl, 10 mM Na_2_HPO_4_, 1.8 mM KH_2_PO_4_ pH 7.4).

*E. coli* cells were incubated for 60 min with lysozyme (100 μg/mL) and were lysed by extensive sonication (25 cycles of 1 min). The sample was centrifuged at 8,000 × *g* for 15 min to obtain the supernatant, which contained the soluble protein fraction. The recombinant protein was purified by affinity chromatography under no denaturing conditions. The soluble fraction was placed in a Glutathione Sepharose× 4B resin column (GE Healthcare®). The resin was washed five times in 1x PBS, and the recombinant protein was cleaved by the addition of thrombin protease (50 U/mL). The purity and size of the recombinant protein were evaluated by running the molecule on 12% SDS-PAGE followed by Coomassie blue staining. *E. coli* cells transformed with pGEX-4 T-3 without an insert for the expression and purification of the protein glutathione S transferase (GST) were used as the experimental control.

### Antibody production

The purified *Pb*MLS was used to produce anti-*Pb*MLS polyclonal antibodies in New Zealand rabbits. The immunization protocol constituted an initial injection of 300 μg of purified recombinant protein in complete Freund’s adjuvant and two subsequent injections of the same amount of the antigen in incomplete Freund’s adjuvant. Each immunization was followed by a 14-day interval. After the fourth immunization, the serum containing the anti-*Pb*MLS polyclonal antibody was collected and stored at −20°C.

### Pull-down assays

A total of 5 mg of each protein extract of *Paracoccidioides Pb*01 mycelium, yeast, yeast secretions and macrophage was incubated with 20 μL of resin bound to GST for 2 h at 4°C under gentle agitation (control). The resin was centrifuged at 200 × *g* for 5 min, and the supernatant was placed into a tube that contained 100 μL of the resin bonded to *Pb*MLS. This mixture was incubated for 3 h at 4°C, with stirring. After this period, the resin was centrifuged at 200 × *g* for 5 min, and the supernatant was discarded. Both resins were washed four times with 1x PBS buffer and subjected to SDS-PAGE on 15% polyacrylamide gel followed by staining with Coomassie Blue (GE Healthcare®).

Separated by SDS-PAGE, the proteins that interacted with *Pb*MLS in the pull-down assay were excised from the gel and identified by MS. Pieces of the gels were soaked in 50 μL of acetonitrile. The solvent was removed under a vacuum and was incubated in 100 mM NH_4_HCO_3_ buffer containing 10 mM 1,4-dithiothreitol for 1 h at 56°C under gentle agitation. The above buffer was removed and replaced by 55 mM iodoacetamide in 100 mM NH_4_HCO_3_ for 45 min at room temperature in the dark. The gel pieces were then subjected to alternating 5 min washing cycles with NH_4_HCO_3_ and acetonitrile, dried down, swollen in 50 μL of 50 mM NH_4_CO_3_ containing 12.5 ng/mL sequencing-grades modified porcine trypsin (Promega, Madison, WI) and incubated at 37°C overnight. The resulting tryptic peptides were extracted by adding 20 μL of 5% v/v acetic acid and removing the solution. This procedure was repeated once. The extracts were pooled, dried under a vacuum and then solubilized in 0.1% v/v trifluoroacetic acid for MS analysis. The proteins of the tryptic digestion samples were analyzed using a MALDI-Synapt MS™ mass spectrometer (Waters-Micromass, Manchester, UK). The peptide mass list obtained for each spectrum was searched using the MASCOT algorithm [14]. Proteins were identified by Peptide Mass Fingerprint (PMF) and/or MS/MS, even considering 1 tryptic cleavage lost, score > 60, 50–100 ppm mass error between theoretical and experimental masses and oxidized methionine as variable modification resulting from in-gel digestion.

### Two-hybrid assays

A cDNA library was obtained using RNA extracted from *Paracoccidioides Pb*01 yeast cells, as described previously [51]. The cDNAs were synthesized and cloned into the prey vector pGADT7 to perform yeast two-hybrid screens using the Matchmaker Two-Hybrid System 3 (Clontech Laboratories, Polo Alto, CA). To screen protein-protein interactions *in vivo* with the MLS, the cDNA encoding *Pb*MLS was sub-cloned into the bait vector pGBKT7. The generation of transformants was obtained by introducing the bait vector into the *Saccharomyces cerevisiae* yeast strain Y187 (MATα, *trp*1-901) and the prey vector into the *S. cerevisiae* strain AH109 (MATα, *leu*2-3).

The experimental protocol was performed according to the Matchmaker GAL4 Two-Hybrid System 3 manual and the Yeast Protocol Handbook (Clontech). Following cell mating, the *S. cerevisiae* diploids that contained the two vectors were selected from plates that contained SD/–Leu/–Trp minimal media. To exclude false-positive clones, the colonies were replicated using high-stringency plates that contained SD–Ade/–His/–Leu/–Trp minimal media. The screening of positive clones was accomplished by detecting the blue/white color of the substrate 5-bromo-4-chloro-3-indolyl-α-D-galactopyranoside (X-α-GAL). Adenine and histidine were the reporter genes that expressed together with lacZ (α-galactosidase reporter gene). A PCR colony assay was performed on the clones using AD-LD 5′ and AD-LD 3′ supplied oligonucleotides for the pGADT7-Rec bait plasmid. The PCR products of the identified transformants were subjected to DNA sequencing using a MegaBACE 1000 sequencer (GE Healthcare®) for automated sequence analysis. Sequence homologies to the genes of interest were performed by searching the GenBank database using the BLAST algorithm [17].

### Construction of protein interaction maps

The Osprey Network Visualization System [25] was used to design a complex interaction network to enable viewing and manipulation [52]. This program uses The GRID protein interaction databases [24] and the *Saccharomyces* Genome Database - SGD [53]. In this way, interaction maps were obtained from pull-down and two-hybrid *Paracoccidioides Pb*01 protein data. The names of the proteins correspond to *S. cerevisiae*, and this correspondence was obtained through analysis of the structural genome databases of *Paracoccidioides Pb*01 [54] and *S. cerevisiae*[23].

### Far-Western blot assays

Far-Western blot assays were conducted as previously described [9]. *Pb*MLS was submitted to SDS-PAGE and blotted onto nylon membrane. After blocking for 4 h with 1.5% (w/v) BSA in 10 mM PBS-milk and washing three times (for 10 min each) in 10 mM triton in PBS (PBS-T), the membranes were incubated with *Paracoccidioides Pb*01 mycelium protein extract (100 μg/mL), yeast cells (100 μg/mL) and macrophage protein extract (100 μg/mL), diluted in PBS-T with 2% BSA for 90 min, and then washed three times (for 10 min each) in PBS-T. The membranes were incubated for 18 h with rabbit IgG anti-enolase, anti-triosephosphate isomerase and anti-actin, respectively, in PBS-T with 2% BSA (1:1000 dilution). The blots were washed with PBS-T and incubated with the secondary antibodies anti-rabbit IgG (1:1000 dilution). The blots were washed with PBS-T and subjected to reaction with alkaline phosphatase. The reaction was developed with 5-bromo-4-chloro-3-indolylphosphate / nitro-bluetetrazolium (BCIP–NBT). The negative control was obtained by incubating *Pb*MLS with anti-enolase, anti-triosephosphate isomerase and anti-actin antibodies, without preincubation with the protein extracts. The positive control was obtained by incubating the *Pb*MLS with the anti-*Pb*MLS antibody, following the reaction as previously described. Another Far-Western blot experiment was performed using the same protocol, but protein extracts of *Paracoccidioides Pb*01 (mycelium, yeast and yeast-secreted) and macrophages were subjected to SDS-PAGE and were blotted onto nylon membrane. The membranes were incubated with *Pb*MLS (100 μg/mL) and subsequently with the primary antibody anti-*Pb*MLS (1:4000 dilution) and the secondary antibody anti-rabbit immunoglobulin (1:1000 dilution). The negative control was obtained by incubating each protein extract with anti-*Pb*MLS antibody, without preincubation with *Pb*MLS.

### Immunofluorescence assays

An immunofluorescence experiment was performed as previously described [55]. J774 A.1 mouse macrophage cells (10^6^ cells/mL) were cultured over cover slips in 6-well plates and were subjected to a recombinant *Pb*MLS binding assay. Mammalian cells were cultured in RPMI supplemented with interferon gamma (1 U/mL). The medium was removed, and the cells were washed 3 times with PBS, fixed for 30 min with cold methanol and air-dried. Either recombinant *Pb*MLS (350 μg/mL) or 1% BSA (w/v, negative control) in PBS was added and incubated with fixed macrophage cells at room temperature for 1 h. After the cells were washed 3 times with PBS, anti-*Pb*MLS antibody (1:1000 dilution) was added. The system was incubated for 1 h at 37 °C and washed 3 times with PBS. The cells were incubated with anti-rabbit IgG coupled to fluoresce in isothiocyanate (FITC; 1:100 dilution) for 1 h. The cells were incubated with 50 μM 4′, 6- diamidino-2-phenylindole (DAPI) for nuclear staining.

### Confocal laser scanning microscopy

A confocal laser scanning microscopy experiment was performed as described by Batista *et al.*[56] and Lenzi *et al*. [57]. A549 cell cultivation and adhesion of the *Paracoccidioides* strain *Pb*18 were performed. The total adhesion (infection and invasion) assays were accomplished in 24 well-plates that contained cover slips at the bottom. In all of the tests, a cellular suspension with 10^6^ cells/mL was standardized. After the tripsinization of the cell suspension, 0.2 mL was removed from the bottle and diluted in 1.8 mL of HAM F12 medium. Cells were counted with a hemocytometer after several dilutions until the appropriate concentration was defined. Later, 0.5 mL of the adjusted cell concentration was placed in each well of the plates and incubated at 36°C for 24 h.

The monolayers were fixed and washed in PBS and permeabilized in 0.5% Triton X-100 for 30 min. After the permeabilization step, the primary antibody anti-*Pb*MLS (1:50 in PBS + 3% skimmed milk + 1% BSA) was added for 1 h, unbound antibody was removed by washing in PBS, and then, Alexa Fluor 594-conjugated antibody goat anti-rabbit IgG (1:400) (1:50 in PBS + 3% skimmed milk + 1% BSA) was added for 1 h, followed by three additional washings with frozen PBS-T before mounting in 90% glycerol in PBS, adjusted to pH 8.5 and containing an anti-fading agent (*p*-phenylenediamine 1 g/L) (Sigma-Aldrich). The specimens were analyzed by laser confocal microscopy using differential interference contrast microscopy (DIC) and fluorescence (LSM 510-META, Zeiss).

### 3D Structures of *Pb*MLS-interacting proteins

The 3D structures of proteins binding to *Pb*MLS (*Pb*MLS-interacting proteins) were initially predicted by the homology modeling method using the modeler algorithm on the ModWeb server [58]. The quality of the structures predicted was measured at NIH-MBI laboratory servers [59] with the ERRAT web server [60]. A Ramachandran plot of each protein was checked/conferred on the RAMPAGE web server [26,61], and Verify 3D was used to evaluate the amino acid environments [62]. The percentages of helical and sheet content were estimated using the 2Struc DSSP server [63] and Helix System [64] for linear representation of the secondary structures.

Molecular Dynamics (MD) simulations of these structures were performed using GROMACS software [27,65] to improve the relaxation and orientation of their side chains and to reproduce the structural stability of the receptor in its native environment [66]. The Particles Mesh Ewald method [67] was used to improve treatment approaches that involve electrostatic interactions with periodic boundary conditions, which were considered in all directions from the box. Initially, the system was neutralized by adding counter ions, and then, it was immediately subjected to minimization using steepest descent energy. The simulations were completed when the tolerance of 1000 kJ/mol was no longer exceeded. The first step in the equilibration of the system was energy relaxation of the solvent for 100 ps (pico seconds); only after this step was the system subjected to MD. With a constant temperature of 300 K, 1 atm pressure, a time-step of 2 fs (femto seconds) and without any restriction of the protein conformations, the simulations were performed for 20 ns (nano seconds) to 60 ns, depending on the protein.

All of the information concerning the trajectory of these times was collected every 5 ps. The equilibration of the trajectory was checked by monitoring the equilibration of the quantities, such as the RMSD of non-hydrogen atoms with respect to the initial structure. Analysis of the total energy, potential energy and kinetic energy were all obtained using GROMACS software. RMSD values between final and template structures also helped to identify the common segments, which corresponds to the structurally conserved region.

The average structure of the entire trajectory was also determined using the g_rms algorithm [68]. The first 10 ns of the trajectory were not used to determine the average structures. All of the water molecules were removed from the selected structures to proceed with the docking simulations in the next step.

### Molecular docking

By using the structures of *Pb*MLS-interacting proteins determined by MD as described above, a global search of protein-protein interactions was performed using GRAMM-X software [69]. The Protein-Protein Docking Web Server v.1.2.0 was used to perform rigid docking. Simulations were performed with no pre-conceived bias toward specific residue interactions, and the best model-structure of each complex (*Pb*MLS + *Pb*MLS-interacting proteins) was selected.

### Refinement of MD

MD simulations of the complexes were performed to improve the orientation of their side chains and to minimize the high-magnitude repulsive interactions between atoms. Short simulations were performed for the complexes defined by the GRAMM-X software, again using GROMACS software, with the same force field and solvent model previously used to define the 3D-structures of each protein. The system was defined by a cubic box with periodic boundary conditions, and a 9 Å cut-off for non-bond interactions was used for electrostatic interactions treated by the Particle Mesh Ewald method. Overlapping water molecules were deleted, and the systems were neutralized by adding counter ions.

Initially, the system was subjected to minimization using steepest descent energy. The simulations were completed when the tolerance of 1000 kJ/mol was no longer exceeded. After minimization, the system was subjected to a 100 ps simulation in the NVT ensemble and then was immediately subjected to a 100 ps simulation in the NPT ensemble. For both stages, T = 300 K, and the thermostat relaxation constant = 0.1 ps; additionally, a Berendsen thermostat, 1 atm pressure, a time-step of 2 fs and position restraint of the complex were used. After that step, the system was subjected to an MD run in the NPT ensemble. The simulations were performed for 1 ns with a constant temperature of 300 K, 1 atm pressure, a time-step of 2 fs and without any restriction on the complex conformations. The structure of the complex used to define the interface region between the proteins was that obtained at the end of the simulations. Fiberdock software [70] was used to estimate the global-energy that was involved in this interface.

## Competing interests

The authors declare that they have no competing interests.

## Authors’ contributions

KMO performed pull-down assays, Far-Western blot assays and immunofluorescence microscopy. BRSN performed two-hybrid assays and prepared samples for confocal microscopy assays. KMO and BRSN prepared the interaction maps. RAS and GOQ performed Molecular Docking and Molecular Dynamics. ARV and MJSMG performed confocal microscopy assays. KMO, BRSN, RAS, MJSMG, JAP, CMAS and MP contributed to the discussion of the data and preparation of the manuscript. MP conceived, designed and coordinated the study. All authors contributed to the discussion of results. All the authors have read and approved the final manuscript.

## Supplementary Material

Additional file 1: Figure S1 Pull-down assays for the determination of *in vitro* interactions between *Pb*MLS and other proteins of *Paracoccidioides*. (**A**) Purification of GST protein (lane 1) and recombinant *Pb*MLS (lane 2) by affinity resin. The proteins detected after the purification of *Pb*MLS were removed from the gel and identified by MS (Additional file 2: Table S1). GST protein was incubated with protein extracts of *Paracoccidioides* mycelium (**B**), yeast (**C**), secretions (**D**) and macrophages (**E**), during which we aimed to remove nonspecific binding proteins (lane 1). After incubation, the supernatant was incubated with *Pb*MLS-GST (purified). The protein complex resulting from this interaction was resolved by SDS-PAGE (lane 2). The proteins numbered were removed from the gel and identified by MS (Additional file 2: Table S1). Click here for file

Additional file 2: Table S1 *Pb*MLS -interacting proteins by using pull-down assays identified by MS. Click here for file

Additional file 3: Table S2 *Pb*MLS-interacting proteins identified by pull-down assays. Click here for file

Additional file 4: Table S3 Gene products interacting with *Pb*MLS by using two-hybrid assay identified by sequencing. Click here for file

Additional file 5: Table S4 *Pb*MLS-interacting proteins already described in the database interactions The GRID indicated in Figure 1. Click here for file

Additional file 6: Table S5 3D Models informations of *Pb*MLS and *Pb*MLS-interacting proteins. Click here for file

Additional file 7: Table S6 Key residues and scores of the protein-protein interaction interface. Click here for file

## References

[B1] Brummer E Castaneda E Restrepo A Paracoccidioidomycosis: an update Clin Microbiol Rev 1993 6 89 117 8472249 10.1128/cmr.6.2.89 PMC358272

[B2] Bernard G Kavakama J Mendes-Giannini MJM Kono A Duarte AJ Shikanai-Yasuda MA Contribution to the natural history of paracocidioidomycosis: identification of primary pulmonary infection in the severe acute form of the disease - a case report Clin Infect Dis 2005 40 1 4 10.1086/426023 15614683

[B3] San-Blas G Niño-Vega G Iturriaga T *Paracoccidioides brasiliensis* and paracoccidioidomycosis: molecular approaches to morphogenesis, diagnosis, epidemiology, taxonomy and genetics Med Mycol 2002 40 225 242 12146752 10.1080/mmy.40.3.225.242

[B4] Coutinho ZF Silva D Lazéra M Petri V Oliveira RM Sasbroza PC Wanke B Paracoccidioidomycosis mortality in Brazil Caderno Saúde Publica 2002 18 1441 1454 10.1590/S0102-311X2002000500037 12244377

[B5] Prado M Silva MB Laurenti R Travassos LR Taborda CP Mortality due to systemic mycoses as a primary cause of death or in association with AIDS in Brazil: a review from 1996 to 2006 Mem Inst Oswaldo Cruz 2009 104 513 521 10.1590/S0074-02762009000300019 19547881

[B6] Bastos KP Bailão AM Borges CL Faria FP Felipe MSS Silva MG Martins WS Fiúza RB Pereira M Soares CMA The transcriptome analysis of early morphogenesis in *Paracoccidioides brasiliensis* mycelium reveals novel and induced genes potentially associated to the dimorphic process BMC Microbiol 2007 10 7 29 10.1186/1471-2180-7-29 PMC1855332 17425801

[B7] Derengowski LS Tavares AH Silva S Procópio LS Felipe MS Silva-Pereira I Upregulation of glyoxylate cycle genes upon *Paracoccidioides brasiliensis* internalization by murine macrophages and *in vitro* nutritional stress condition Med Mycol 2008 46 125 134 10.1080/13693780701670509 18324491

[B8] Zambuzzi-Carvalho PF Cruz AHS Santos-Silva LK Goes AM Soares CMA Pereira M The malate synthase of *Paracoccidioides brasiliensis Pb*01 is required in the glyoxylate cycle and in the allantoin degradation pathway Med Mycol 2009 1 1 11 10.3109/13693780802609620 19888806

[B9] Neto BRS Silva JF Mendes-Giannini MJS Lenzi HL Soares CMA Pereira M The malate synthase of *Paracoccidioides brasiliensis* is a linked surface protein that behaves as an anchorless adhesion BMC Microbiol 2009 9 272 284 10.1186/1471-2180-9-272 20034376 PMC2807876

[B10] Auerbach D Thaminy S Hottiger MO Stagljar I The post-genomic era of interactive proteomics: facts and perspectives Proteomics 2002 2 611 623 10.1002/1615-9861(200206)2:6<611::AID-PROT611>3.0.CO;2-Y 12112840

[B11] Vikis HG Guan KL Glutathione-S-transferase-fusion based assays for studying protein-protein interactions Methods Mol Biol 2004 261 175 186 15064458 10.1385/1-59259-762-9:175

[B12] Rezende TC Borges CL Magalhães AD de Sousa MV Ricart CA Bailão AM Soares CM A quantitative view of the morphological phases of *Paracoccidioides brasiliensis* using proteomics J Proteomics 2011 75 572 587 10.1016/j.jprot.2011.08.020 21920475

[B13] Ellis RJ van der Vies SM Molecular chaperones Annu Rev Biochem 1991 60 321 347 10.1146/annurev.bi.60.070191.001541 1679318

[B14] MASCOT algorithm http://www.matrixscience.com

[B15] UniProt databases http://www.uniprot.org/

[B16] MIPS http://mips.helmholtz-muenchen.de/genre/proj/yeast/

[B17] BLAST algorithm http://www.ncbi.nlm.nih.gov

[B18] PEDANT 3 database http://pedant.helmholtz-muenchen.de/index.jsp

[B19] Costanzo M Baryshnikova A Bellay J Kim Y Spear ED Sevier CS Ding H Koh JL Toufighi K Mostafavi S Prinz J St Onge RP VanderSluis B Makhnevych T Vizeacoumar FJ Alizadeh S Bahr S Brost RL Chen Y Cokol M Deshpande R Li Z Lin ZY Liang W Marback M Paw J San Luis BJ Shuteriqi E Tong AH van Dyk N The genetic landscape of a cell Science 2010 327 425 431 10.1126/science.1180823 20093466 PMC5600254

[B20] Tong A Boone C Synthetic genetic array analysis in *Saccharomyces cerevisiae* Meth Mol Biol 2006 313 171 192 10.1385/1-59259-958-3:171 16118434

[B21] Tong AH Lesage G Bader GD Ding H Xu H Xin X Young J Berriz GF Brost RL Chang M Chen Y Cheng X Chua G Friesen H Goldberg DS Haynes J Humphries C He G Hussein S Ke L Krogan N Li Z Levinson JN Lu H Ménard P Munyana C Parsons AB Ryan O Tonikian R Roberts T Global mapping of the yeast genetic interaction network Science 2004 303 808 813 10.1126/science.1091317 14764870

[B22] Collins SR Miller KM Maas NL Roguev A Fillingham J Chu CS Schuldiner M Gebbia M Recht J Shales M Ding H Xu H Han J Ingvarsdottir K Cheng B Andrews B Boone C Berger SL Hieter P Zhang Z Brown GW Ingles CJ Emili A Allis CD Toczyski DP Weissman JS Greenblatt JF Krogan NJ Functional dissection of protein complexes involved in yeast chromosome biology using a genetic interaction map Nature 2007 446 806 810 10.1038/nature05649 17314980

[B23] Structural genome databases of *Saccharomyces cerevisiae* http://www.broadinstitute.org/annotation/genome/saccharomyces_cerevisiae

[B24] The GRID protein interaction databases http://thebiogrid.org/

[B25] Osprey network visualization system - version 1.2.0 http://biodata.mshri.on.ca/osprey/servlet/Index

[B26] RAMPAGE web server http://mordred.bioc.cam.ac.uk/~rapper/rampage.php

[B27] GROMACS software http://www.gromacs.org/

[B28] Cho S Park SG Lee DH Park BC Protein-protein interaction networks: from interactions to networks J Biochem Mol Biol 2004 37 45 52 10.5483/BMBRep.2004.37.1.045 14761302

[B29] Felipe MS Andrade RV Arraes FB Nicola AM Maranhão AQ Torres FA Silva-Pereira I Poças-Fonseca MJ Campos EG Moraes LM Andrade PA Tavares AH Silva SS Kyaw CM Souza DP Pereira M Jesuíno RS Andrade EV Parente JA Oliveira GS Barbosa MS Martins NF Fachin AL Cardoso RS Passos GA Almeida NF Walter ME Soares CM Carvalho MJ Brígido MM Transcriptional profiles of the human pathogenic fungus *Paracoccidioides brasiliensis* in mycelium and yeast cells J Biol Chem 2005 280 24706 24714 10.1074/jbc.M500625200 15849188

[B30] Gietl C Malate dehydrogenase isoenzymes: cellular locations and role in the flow of metabolites between the cytoplasm and cell organelles Biochim Biophys Acta 1992 1100 217 234 10.1016/0167-4838(92)90476-T 1610875

[B31] Hanks SK Quinn AM Hunter T The protein kinase family: conserved features and deduced phylogeny of the catalytic domains Science 1998 241 42 52 3291115 10.1126/science.3291115

[B32] Silva AH Brock M Zambuzzi-Carvalho PF Santos-Silva LK Troian RF Góes AM Soares CMA Pereira M Phosphorylation is the major mechanism regulating isocitrate lyase activity in *Paracoccidioides brasiliensis* yeast cells FEBS Journal 2011 278 2318 2332 10.1111/j.1742-4658.2011.08150.x 21535474

[B33] Vallejo MC Nakayasu ES Matsuo AS Sobreira TJP Longo LVG Ganiko L Almeida IC Puccia R Vesicle and vesicle-free extracellular proteome of *Paracoccidioides brasiliensis:* Comparative analysis with other pathogenic fungi J Proteome Res 2012 11 1676 1685 10.1021/pr200872s 22288420 PMC3319080

[B34] Bonin-Debs AL Boche I Gille H Brinkmann U Development of secreted proteins as biotherapeutic agents Expert Opin Biol Ther 2004 4 551 558 10.1517/14712598.4.4.551 15102604

[B35] Tjalsma H Antelmann H Jongbloed Proteomics of protein secretion by *Bacillus subtilis:* separating the “secrets” of the secretome Microbiol and Mol Biol Rev 2004 68 207 233 10.1128/MMBR.68.2.207-233.2004 15187182 PMC419921

[B36] Weber SS Parente AFA Borges CL Parente JA Bailão AM Soares CMA Analysis of the secretomes of *Paracoccidioides* mycelia and yeast cells PLoS ONE 2012 7 e52470 10.1371/journal.pone.0052470 23272246 PMC3525554

[B37] Marchais V Kempf M Licznar P Lefrançois C Bouchara JP Robert R Cottin J DNA array analysis of *Candida albicans* gene expression in response to adherence to polystyrene FEMS Microbiol 2005 245 25 32 10.1016/j.femsle.2005.02.014 15796975

[B38] González A Gomez BL Diez S Hernandez O Restrepo A Hamilton AJ Cano LE Purification and partial characterization of a *Paracoccidioides brasiliensis* protein with capacity to bind to extracellular matrix proteins Infect Immun 2004 73 2486 2495 15784595 10.1128/IAI.73.4.2486-2495.2005 PMC1087412

[B39] Barbosa MS Bao SN Andreotti PF De Faria FP Felipe MSS Feitosa LS Mendes-Giannini MJS Soares CMA Glyceraldehyde-3-phosphate dehydrogenase of *Paracoccidioides brasiliensis* is a cell surface protein involved in fungal adhesion to extracellular matrix proteins and interaction with cells Infect Immun 2006 74 382 389 10.1128/IAI.74.1.382-389.2006 16368993 PMC1346668

[B40] Mendes-Giannini MJS Hanna SA da Silva JL Andretti PF Vicentini LR Bernard G Lenzi HL Soares CP Invasion of epithelial mammalian cells by *Paracoccidioides brasiliensis* leads to cytoskeletal rearrangement and apoptosis of the host cell Microbes Infect 2004 6 882 891 10.1016/j.micinf.2004.05.005 15310464

[B41] Castro NDS Barbosa MS Maia ZA Báo SN Felipe MS Santana JM Mendes-Giannini MJS Pereira M Soares CMA Characterization of *Paracoccidioides brasiliensis* PbDfg5p, a cell-wall protein implicated in filamentous growth Yeast 2008 25 141 154 10.1002/yea.1574 18098122

[B42] Pereira LA Bao SN Barbosa MS Silva JL Felipe MS Santana JM Mendes-Giannini MJS Soares CMA Analysis of the *Paracoccidioides brasiliensis* triosephosphate isomerase suggests the potentialfor adhesin function FEMS Yeast Res 2007 7 1381 1388 10.1111/j.1567-1364.2007.00292.x 17714474

[B43] Donofrio FC Calil AC Miranda ET Almeida AM Benard G Soares CP Nogueira SV Soares CMA Mendes-Giannini MJS Enolase from *Paracoccidioides brasiliensis:* isolation and identification as fibronectin-binding protein J Med Microbiol 2009 58 706 713 10.1099/jmm.0.003830-0 19429745

[B44] Coelho Neto J Agero U Oliveira DC Gazzinelli RT Mesquita ON Real-time measurements of membrane surface dynamics on macrophages and the phagocytosis of *Leishmania* parasites Exp Cell Res 2005 303 207 217 10.1016/j.yexcr.2004.09.002 15652336

[B45] Pereanez JA Gómez ID Patino AC Relationship between the structure and the enzymatic activity of crotoxin complex and its phospholipase A2 subunit: An *in silico* approach J Mol Graph and Model 2012 35 36 42 22481077 10.1016/j.jmgm.2012.01.004

[B46] Burger AM Seth AK The ubiquitin-mediated protein degradation pathway in cancer: therapeutic implications Eur J Cancer 2004 40 2217 2229 10.1016/j.ejca.2004.07.006 15454246

[B47] Jeferry CJ Mass spectrometry and the search for moonlighting proteins Mass Spectrom Rev 2005 24 772 782 10.1002/mas.20041 15605385

[B48] Borges CL Pereira M Felipe MSS Faria FP Gomez FJ Deepe GS Soares CMA The antigenic and catalytically active formamidase of *Paracoccidioides brasiliensis*: protein characterization, cDNA and gene cloning, heterologous expression and functional analysis of the recombinant protein Microbes Infect 2005 7 66 77 10.1016/j.micinf.2004.09.011 15716068

[B49] Bradford MM A rapid and sensitive method for the quantitation of microgram quantities of protein utilizing the principle of protein-dye binding Anal Biochem 1976 72 248 254 10.1016/0003-2697(76)90527-3 942051

[B50] Cell Bank in Rio de Janeiro, Brazil http://b200.nce.ufrj.br/bcrj/index.php?option=com_content&task=view&id=10&Itemid=30

[B51] Borges CL Parente JA Barbosa MS Santana JM Báo SN Sousa MV Soares CMA Detection of a homotetrameric structure and protein-protein interactions of *Paracoccidioides brasiliensis* formamidase lead to new functional insights FEMS Yeast Res 2010 10 104 113 10.1111/j.1567-1364.2009.00594.x 20002196

[B52] Breitkreutz BJ Stark C Tyers M Osprey: a network visualization system Genome Biol 2003 4 22 10.1186/gb-2003-4-3-r22 PMC153462 12620107

[B53] *Saccharomyces* Genome Database – SGD http://www.yeastgenome.org/

[B54] Structural genome databases of *Paracoccidioides brasiliensis* http://www.broadinstitute.org/annotation/genome/paracoccidioides_brasiliensis

[B55] Bailão AM Nogueira SV Bonfim SMRC Castro KP da Silva JF Mendes-Giannini MJS Pereira M Soares CMA Comparative transcriptome analysis of *Paracoccidioides brasiliensis* during *in vitro* adhesion to type I collagen and fibronectin: identification of potential adhesins Res Microbiol 2012 163 182 191 10.1016/j.resmic.2012.01.004 22306611

[B56] Batista WL Matsuo AL Ganiko L Barros TF Veiga TR Freymüller E Puccia R The *PbMDJ1* gene belongs to a conserved *MDJ1/LON* locus in thermodimorphic pathogenic fungi and encodes a heat shock protein that localizes to both the mitochondria and cell wall of *Paracoccidioides brasiliensis* Eukaryot Cell 2006 5 379 390 10.1128/EC.5.2.379-390.2006 16467478 PMC1405898

[B57] Lenzi HL Pelajo-Machado M Vale BS Panasco MS Microscopia de Varredura Laser Confocal: Princípios e Aplicações Biomédicas Newslab 1996 16 62 71

[B58] Eswar N John B Mirkovic N Fiser A Ilyin VA Pieper U Stuart AC Marti-Renom MA Madhusudhan MS Yerkovich B Tools for comparative protein structure modeling and analysis Nucleic Acids Res 2003 31 3375 3380 10.1093/nar/gkg543 12824331 PMC168950

[B59] NIH-MBI laboratory servers http://nihserver.mbi.ucla.edu

[B60] Colovos C Yeates TO Verification of protein structures: patterns of nonbonded atomic interactions Protein Sci 1993 2 1511 1519 10.1002/pro.5560020916 8401235 PMC2142462

[B61] Lovell SC Davis IW Arendall WB III Bakker PIW Word JM Prisant MG Richardson JS Richardson DC Structure validation by Calpha geometry: phi, psi and Cbeta deviation Proteins Struct Funct Genet 2002 50 437 450 12557186 10.1002/prot.10286

[B62] Luthy R Bowie JU Eisenberg D Assessment of protein models with three-dimensional profiles Nature 1992 356 83 85 10.1038/356083a0 1538787

[B63] Kabsch W Sander C Dictionary of protein secondary structure: pattern recognition of hydrogen-bonded and geometrical feature Biopolymers 1983 22 2577 2637 10.1002/bip.360221211 6667333

[B64] Helix System http://helix.nih.gov

[B65] Okimoto N Futatsugi N Fuji H Suenaga A Morimoto G Yanai R Ohno Y Narumi T Tai M High-performance drug discovery: computational screening by combining docking and molecular dynamics simulations PLoS Comput Biol 2009 5 e1000528 10.1371/journal.pcbi.1000528 19816553 PMC2746282

[B66] Sakkiah S Thangapandian S Woo-Lee K Pharmacophore modeling, molecular docking, and molecular dynamics simulation approaches for identifying new lead compounds for inhibiting aldose reductase J Mol Model 2012 2 2249 2747 10.1007/s00894-011-1247-5 22249747

[B67] Darden T York D Pederson L Particle mesh Ewald: An N·log(N) method for Ewald sums in large systems J Chem Phys 1993 98 10089 10092 10.1063/1.464397

[B68] Maiorov VN Crippen GM Size-independent comparison of protein three- dimensional structures Proteins Struct Funct Genet 1995 22 273 283 10.1002/prot.340220308 7479700

[B69] Tovchigrechko A Vakser IA GRAMM-X public web server for protein-protein docking Nucleic Acids Res 2006 34 310 314 10.1093/nar/gkl206 PMC1538913 16845016

[B70] Mashiach E Nussinov R Wolfson HJ FiberDock: flexible induced-fit backbone refinement in molecular docking Proteins 2009 78 1503 1519 20077569 10.1002/prot.22668 PMC4290165

