# Supplementary figures and images for: Intermolecular interactions of the malate synthase of Paracoccidioides spp
Source: BMC Microbiol. 2013 May 14;13:107. doi: 10.1186/1471-2180-13-107 (PMC3771410; doi:10.1186/1471-2180-13-107)

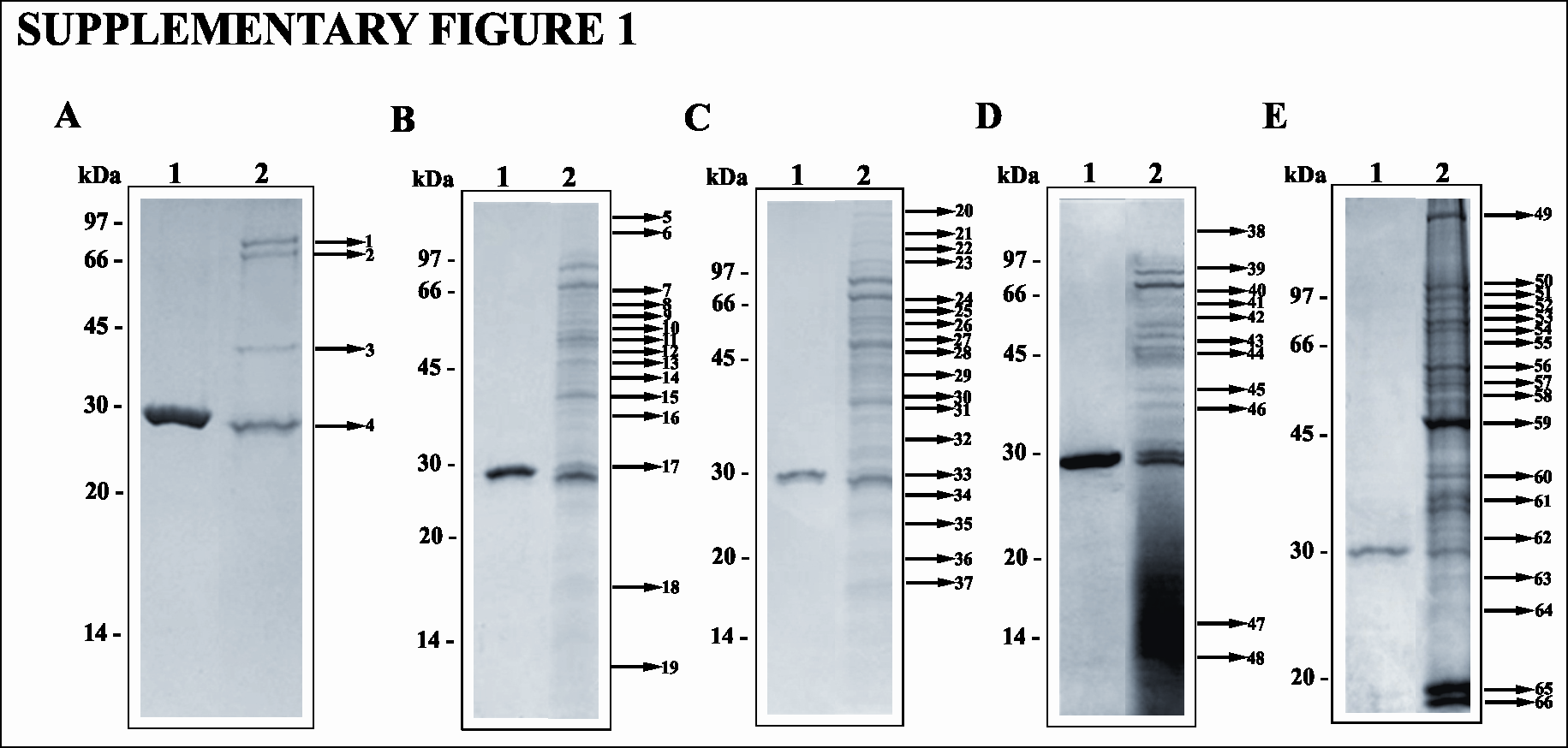

Supplement: Additional file 1: Figure S1 — Pull-down assays for the determination of in vitro interactions between PbMLS and other proteins of Paracoccidioides. (A) Purification of GST protein (lane 1) and recombinant PbMLS (lane 2) by affinity resin. The proteins detected after the purification of PbMLS were removed from the gel and identified by MS (Additional file 2: Table S1). GST protein was incubated with protein extracts of Paracoccidioides mycelium (B), yeast (C), secretions (D) and macrophages (E), during which we aimed to remove nonspecific binding proteins (lane 1). After incubation, the supernatant was incubated with PbMLS-GST (purified). The protein complex resulting from this interaction was resolved by SDS-PAGE (lane 2). The proteins numbered were removed from the gel and identified by MS (Additional file 2: Table S1). [file 1471-2180-13-107-S1.tif]
