# Supplementary material for: Intermolecular interactions of the malate synthase of Paracoccidioides spp
Source: BMC Microbiol. 2013 May 14;13:107. doi: 10.1186/1471-2180-13-107 (PMC3771410; doi:10.1186/1471-2180-13-107)
Supplement: Additional file 2: Table S1 — PbMLS -interacting proteins by using pull-down assays identified by MS. [file 1471-2180-13-107-S2.docx]

**Additional file 2: Table S1 – *Pb*MLS -interacting proteins by using pull-down assays identified by MS.**

| **Gel** | **Spot Number** | **Protein** | **Accession Number^*^** | **Score** | **Coverage** | **Theoretical Molecular Weight (kDa)** | **Experimental Molecular Weight**  **(kDa)** | **Function^+^** |
| --- | --- | --- | --- | --- | --- | --- | --- | --- |
|  | 1 | Malate synthase | PAAG_04542 | 258 | 61% | 61,02 | 86,40^**^ | --- |
| A | 2 | Malate synthase | PAAG_04542 | 398 | 54% | 61,02 | 86,40^**^ | --- |
|  | 3 | Membrane protein F | [gi\|258690805](http://www.matrixscience.com/cgi/protein_view.pl?file=../data/20120228/FtoriaTtL.dat&hit=gi%7c258690805&db_idx=1&px=1&ave_thresh=36&_ignoreionsscorebelow=0&report=0&_sigthreshold=0.05&_msresflags=1025&_msresflags2=2&percolate=-1&percolate_rt=0) | 126 | 11% | 39,32 | 47,28 | --- |
|  | 4 | Glutathione S- transferase | [gi\|121697](http://www.matrixscience.com/cgi/protein_view.pl?file=../data/20120228/FtoriacOO.dat&hit=gi%7c121697&db_idx=1&px=1&ave_thresh=56&_ignoreionsscorebelow=0&report=0&_sigthreshold=0.05&_msresflags=1025&_msresflags2=2&percolate=-1&percolate_rt=0) | 274 | 73% | 25,71 | 25,50 | --- |
|  |  |  |  |  |  |  |  |  |
|  | 5 | Fatty acid synthase - subunit beta | PAAG_01524 | 154 | 25% | 232,02 | 231,58 | Fatty acid metabolism |
|  | 6 | Mitochondrial protease | PAAG_00705 | 84 | 60% | 118,59 | 118,23 | Protein metabolism |
|  | 7 | Cobalamin synthase | PAAG_07626 | 233 | 53% | 87,30 | 87,14 | Biosynthesis of methionine |
|  | 8 | Heat shock protein  60 kDa | PAAG_08059 | 88 | 62% | 62,26 | 62,14 | Transport and protein folding |
|  | 10 | Peroxisomal catalase | PAAG_01454 | 87 | 50% | 57,65 | 57,25 | Response to oxidative stress |
|  | 9 | ATPase alpha subunit | PAAG_04820 | 81 | 58% | 55,04 | 55,91 | Energy cell |
| B | 11 | Aldehyde dehydrogenase | PAAG_05249 | 202 | 30% | 54,55 | 54,20 | Biogenesis of cellular components |
|  | 12 | 2-Methylcitrate  synthase | PAAG_04550 | 152 | 55% | 51,51 | 51,50 | Energy |
|  | 13 | Enolase | PAAG_00771 | 422 | 61% | 47,41 | 47,66 | Carbohidrate metabolism |
|  | 14 | Elongation factor 1-gamma | PAAG_03556 | 337 | 58% | 45,88 | 45,80 | Protein synthesis |
|  | 15 | Fructose 1,6 bisphosphate aldolase | PAAG_01995 | 138 | 57% | 39,72 | 39,64 | Glycolysis /  gluconeogenesis |
|  | 16 | Malate dehydrogenase | PAAG_00053 | 185 | 70% | 36,02 | 35,99 | Metabolism |
|  | 17 | Nucleic acid-binding protein | PAAG_04814 | 60 | 24% | 30,40 | 30,37 | Processing of nucleic acids |
|  | 18 | Nucleoside diphosphate kinase | PAAG_04291 | 159 | 39% | 16,87 | 16,78 | Nucleotide metabolism |
|  | 19 | Cytochrome C | PAAG_06268 | 118 | 77% | 12,28 | 12,18 | Energy |
|  |  |  |  |  |  |  |  |  |
|  | 20 | Fatty acid synthase - subunit beta | PAAG_01524 | 136 | 33% | 232,02 | 231,58 | Fatty acid metabolism |
|  | 21 | Glutamate dehydrogenase | PAAG_01002 | 166 | 28% | 125,04 | 124,62 | Amino acid metabolism |
|  | 22 | Elongation factor 2 | PAAG_00594 | 139 | 32% | 92,69 | 92,25 | Protein synthesis |
|  | 23 | Cobalamin synthase | PAAG_07626 | 175 | 66% | 87,30 | 87,14 | Biosynthesis of methionine |
|  | 24 | Heat shock protein  60 kDa | PAAG_08059 | 220 | 68% | 62,26 | 62,14 | Transport and protein folding |
| C | 25 | Peroxisomal catalase | PAAG_01454 | 74 | 38% | 57,65 | 57,25 | Response to oxidative stress |
|  | 26 | ATPase alpha subunit | PAAG_04820 | 111 | 57% | 55,04 | 55,91 | Energy |
|  | 27 | 2-Methylcitrate  synthase | PAAG_04550 | 131 | 53% | 51,51 | 51,50 | Energy |
|  | 28 | Enolase | PAAG_00771 | 462 | 52% | 47,41 | 47,66 | Metabolism |
|  | 29 | Elongation factor 1-gamma | PAAG_03556 | 284 | 57% | 45,88 | 45,80 | Protein synthesis |
|  | 30 | Fructose 1,6 bisphosphate aldolase | PAAG_01995 | 223 | 53% | 39,76 | 39,64 | Glycolysis /  gluconeogenesis |
|  | 31 | Glyceraldehyde-3-phosphate dehydrogenase | PAAG_08468 | 231 | 73% | 36,61 | 36,47 | Glycolysis /  gluconeogenesis |
|  | 32 | Malate dehydrogenase | PAAG_00053 | 86 | 52% | 36,02 | 35,99 | Metabolism |
|  | 33 | 40S ribosomal protein S3 | PAAG_01785 | 163 | 69% | 29,76 | 29,62 | Protein synthesis |
|  | 34 | Triosephosphate isomerase | PAAG_02585 | 124 | 20% | 27,15 | 27,07 | Glycolysis /  gluconeogenesis |
|  | 35 | 60S ribosomal protein L18 | PAAG_07955 | 106 | 50% | 21,11 | 21,13 | Protein synthesis |
|  | 36 | Nucleoside diphosphate kinase | PAAG_04291 | 114 | 23% | 16,87 | 16,78 | Nucleotide metabolism |
|  | 37 | Hypothetical protein | PAAG_03664 | 94 | 85% | 16,74 | 16,65 | -- |
|  |  |  |  |  |  |  |  |  |
|  | 38 | Aminopeptidase | PAAG_03279 | 145 | 49% | 108,51 | 103,16 | Peptide metabolism |
|  | 39 | Cobalamin synthase | PAAG_07626 | 176 | 51% | 87,30 | 87,14 | Biosynthesis of methionine |
|  | 40 | Heat shock protein  70 kDa | PAAG_08003 | 355 | 64% | 70,91 | 70,80 | Transport and protein folding |
|  | 41 | 2-methylcitrate dehydratase | PAAG_04559 | 258 | 31% | 62,33 | 61,85 | Energy |
|  | 42 | Pyruvate kinase | PAAG_06380 | 159 | 70% | 59,48 | 59,01 | Glycolysis /  gluconeogenesis |
| D | 43 | 2-methylcitrate  synthase | PAAG_04550 | 343 | 53% | 51,51 | 51,50 | Energy |
|  | 44 | Adenosyl-homocysteinase | PAAG_02859 | 240 | 62% | 49,51 | 48,49 | Amino acid metabolism |
|  | 45 | Malate dehydrogenase | PAAG_00053 | 118 | 28% | 36,02 | 35,99 | Metabolism |
|  | 46 | Nucleotide pyrophosphorylase | PAAG_08856 | 92 | 37% | 33,85 | 33,44 | Nucleotide metabolism |
|  | 47 | Nucleoside diphosphate kinase | PAAG_04291 | 74 | 32% | 16,87 | 16,78 | Nucleotide metabolism |
| 48 | Cytochrome C | PAAG_06268 | 175 | 39% | 12,28 | 12,18 | Energy |  |
|  |  |  |  |  |  |  |  |  |
|  | 49 | Myosin-9 | gi\|114326446 | 367 | 59% | 227,42 | 226,43 | Structure and cytoskeletal |
|  | 50 | Alpha-actinin-4 | gi\|11230802 | 161 | 45% | 105,36 | 105,00 | Endocytosis and cytoskeletal |
|  | 51 | Endoplasmin | gi\|6755863 | 469 | 24% | 92,70 | 92,49 | Cellular structure |
|  | 52 | Heat shock protein  84 kDa | gi\|194027 | 177 | 45% | 83,52 | 83,24 | Transport and protein folding |
|  | 53 | Glucose-regulated protein | gi\|1304157 | 162 | 37% | 72,52 | 72,47 | Glucose metabolism |
|  | 54 | Heat shock protein  70 kDa | gi\|309319 | 189 | 33% | 71,02 | 70,85 | Transport and protein folding |
| E | 55 | Plastin-2 | gi\|31543113 | 151 | 36% | 70,73 | 70,16 | Cellular  structure |
|  | 56 | Heat shock protein  60 kDa | gi\|76779273 | 121 | 65% | 59,55 | 59,43 | Transport and protein folding |
|  | 57 | Vimentin | gi\|31982755 | 130 | 67% | 53,71 | 53,70 | Cellular structure |
|  | 58 | Tubulin beta-5 chain | gi\|7106439 | 174 | 57% | 50,09 | 49,68 | Cytoskeletal and cell cycle |
|  | 59 | Gamma-actin | gi\|809561 | 337 | 34% | 41,33 | 41,02 | Structure and cytoskeletal |
|  | 60 | Fructose 1,6 bisphosphate aldolase | gi\|6671539 | 199 | 60% | 39,78 | 39,36 | Glycolysis /  gluconeogenesis |
|  | 61 | Beta-actin | gi\|49868 | 404 | 28% | 39,44 | 39,19 | Structure and cytoskeletal |
|  | 62 | Prohibitin | gi\|6679299 | 189 | 55% | 29,85 | 29,83 | Anti-proliferative activity |
|  | 63 | 60S ribosomal protein – L10 | gi\|149259021 | 94 | 25% | 25,06 | 24,62 | Protein synthesis |
|  | 64 | 40S ribosomal protein – S27 | gi\|13195690 | 104 | 24% | 18,28 | 17,95 | Protein synthesis |
|  | 65 | Histone H2A | gi\|387182 | 170 | 36% | 14,23 | 14,18 | Processing of DNA |
|  | 66 | Histone H2B | gi\|13386452 | 183 | 47% | 13,98 | 14,00 | Processing of DNA |

*****Accession number by using MASCOT algorithm [14] and structural genome database of *Paracoccidioides* [54].

PAAG refers to *Paracoccidioides* and gi refers to other microorganisms.

******Molecular weight of the Malate synthase considering 25,5 kDa of GST.

^+^Function defined on the basis of information contained in the database UniProt [15] and MIPS [16].

A, B, C, D and E refere to gels from Additional file 1: Figure S1.
