# Supplementary material for: Intermolecular interactions of the malate synthase of Paracoccidioides spp
Source: BMC Microbiol. 2013 May 14;13:107. doi: 10.1186/1471-2180-13-107 (PMC3771410; doi:10.1186/1471-2180-13-107)
Supplement: Additional file 3: Table S2 — PbMLS-interacting proteins identified by pull-down assays. [file 1471-2180-13-107-S3.docx]

**Additional file 3: Table S2 - *Pb*MLS-interacting proteins identified by pull-down assays.**

| **Protein *Paracoccidioides/ Mus musculus*** | **Protein**  ***S. cerevisae*** | **Pull-down assays** | | | |
| --- | --- | --- | --- | --- | --- |
|  |  | **Mycelium** | **Yeast** | **Yeast-Secreted** | **Macrophage** |
|  |  |  |  |  |  |
| Fatty acid synthase subunit beta (PAAG_01524) | Acetyl transferase  (YKL182W) | **X** | **X** |  |  |
| Mitochondrial protease  (PAAG_00705) | Metalloprotease  (YDR430C) | **X** |  |  |  |
| Cobalamin synthase**^●^  (PAAG_07626) | Homocysteine​​methyltransferase  (YER091C) | **X** | **X** | **X** |  |
| Heat shock protein  60 kDa**^●^  (PAAG_08059/*Mus musculus*) | Chaperonin  (YLR259C) | **X** | **X** |  | **X** |
| Peroxisomal catalase  (PAAG_01454) | Catalase A  (YDR256C) | **X** | **X** |  |  |
| ATPase alpha subunit  (PAAG_04820) | ATPase alpha subunit  (YBL099W) | **X** | **X** |  |  |
| Aldehyde dehydrogenase**^●^  (PAAG_05249) | Aldehyde dehydrogenase  (YER073W) | **X** |  |  |  |
| 2-Methylcitrate synthase**^+●^  (PAAG_04550) | Citrate synthase  (YNR001C) | **X** | **X** | **X** |  |
| Enolase**^+●^  (PAAG_00771) | Enolase  (YGR254W) | **X** | **X** |  |  |
| Elongation factor 1 gamma  (PAAG_03556) | Elongation factor 1 gamma  (YKL081W) | **X** | **X** |  |  |
| Fructose 1,6 bisphosphate aldolase**^+●^  (PAAG_01995/*Mus musculus*) | Aldolase  (YKL060C) | **X** | **X** |  | **X** |
| Malate dehydrogenase**^+●^  (PAAG_00053) | Malate  dehydrogenase  (YDL078C) | **X** | **X** | **X** |  |
| Nucleic acid-binding protein**^●^  (PAAG_04814) | Protein nuclear localization  (YGR159C) | **X** |  |  |  |
| Nucleoside diphosphate kinase**^●^  (PAAG_04291) | Nucleoside diphosphate kinase (YKL067W) | **X** | **X** | **X** |  |
| Cytochrome C^●^  (PAAG_06268) | Iso2- Cytochrome C  (YEL039C) | **X** |  | **X** |  |
| Glutamate dehydrogenase  (PAAG_01002) | Glutamate dehydrogenase  (YDL215C) |  | **X** |  |  |
| Elongation factor2  (PAAG_00594) | Elongation factor2  (YDR385W) |  | **X** |  |  |
| Glyceraldehyde-3-phosphate dehydrogenase**^+^  (PAAG_08468) | Glyceraldehyde-3-phosphate dehydrogenase  (YGR192C) |  | **X** |  |  |
| 40S ribosomal  protein S3  (PAAG_01785) | 40S ribosomal  protein S3  (YNL178W) |  | **X** |  |  |
| Triosephosphate isomerase**^+●^  (PAAG_02585) | Triosephosphate  isomerase  (YDR050C) |  | **X** |  |  |
| 60S ribosomal protein L18**^●^  (PAAG_07955) | 60S ribosomal  protein S27  (YNL301C) |  | **X** |  |  |
|  |  |  |  |  |  |
| Aminopeptidase  (PAAG_03279) | Aminopeptidase  (YHR047C) |  |  | **X** |  |
| Heat shock protein  70 kDa (PAAG_08003/*Mus musculus*) | Heat shock protein  70 kDa  (YLL024C) |  |  | **X** | **X** |
| 2-Methylcitrate dehydratase (PAAG_04559) | 2-Methylcitrate dehydratase  (YPR002W) |  |  | **X** |  |
|  |  |  |  |  |  |
| Pyruvate kinase  (PAAG_06380) | Pyruvate kinase  (YAL038W) |  |  | **X** |  |
| Adenosyl-homocysteinase  (PAAG_02859) | S-Adenosyl-L-homocysteine hydrolase  (YER043C) |  |  | **X** |  |
| Nucleotide pyrophosphorylase  (PAAG_08856) | Phosphoribosyltransferase  (YFR047C) |  |  | **X** |  |
| Hypothetical protein**^●^ (PAAG_03664) | --- |  | **X** |  |  |
| Myosin 9**  (*Mus musculus*) | --- |  |  |  | **X** |
| Alpha actinin-4**  (*Mus musculus*) | --- |  |  |  | **X** |
|  |  |  |  |  |  |
| Endoplasmin  (*Mus musculus*) | --- |  |  |  | **X** |
|  |  |  |  |  |  |
| Heat shock protein  84 kDa**  (*Mus musculus*) | --- |  |  |  | **X** |
|  |  |  |  |  |  |
| Glucose-regulated protein  (*Mus musculus*) | --- |  |  |  | **X** |
|  |  |  |  |  |  |
| Plastin-2  (*Mus musculus*) | --- |  |  |  | **X** |
| Vimentin  (*Mus musculus*) | --- |  |  |  | **X** |
| Tubulin beta-5 chain**  (*Mus musculus*) | --- |  |  |  | **X** |
|  |  |  |  |  |  |
| Gamma actin**^+^  (*Mus musculus*) | --- |  |  |  | **X** |
|  |  |  |  |  |  |
| Beta actin**  (*Mus musculus*) | --- |  |  |  | **X** |
| Prohibitin  (*Mus musculus*) | --- |  |  |  | **X** |
| 60S ribosomal protein L10 (*Mus musculus*) | --- |  |  |  | **X** |
| 40S ribosomal protein  S27 (*Mus musculus*) | --- |  |  |  | **X** |
| Histone H2A  (*Mus musculus*) | --- |  |  |  | **X** |
|  |  |  |  |  |  |
| Histone H2B  (*Mus musculus*) | --- |  |  |  | **X** |

Data were obtained from the databases: *Saccharomyces* Genome Database – SGD [53] and structural genome databases of *Paracoccidioides* and *S. cerevisiae* [54, 23].

**Interactions confirmed by Far-Western blot experiments.

^+^Interactions confirmed by *in silico* experiments.

^●^Proteins identified by using multiple methods.
