# Supplementary material for: Intermolecular interactions of the malate synthase of Paracoccidioides spp
Source: BMC Microbiol. 2013 May 14;13:107. doi: 10.1186/1471-2180-13-107 (PMC3771410; doi:10.1186/1471-2180-13-107)
Supplement: Additional file 4: Table S3 — Gene products interacting with PbMLS by using two-hybrid assay identified by sequencing. [file 1471-2180-13-107-S4.docx]

**Additional file 4: Table S3 - Gene products interacting with *Pb*MLS by using two-hybrid assay identified by sequencing.**

| **Gene product** | **Functional classification^a^** | ***Paracoccidioides*^b^** | ***S. cerevisiae*^c^** |
| --- | --- | --- | --- |
| Vacuolar transporte rchaperone3 | Cellular Transport | PAAG_07987 | YPL019C  Vacuolar transporter chaperone2 |
| GTP-binding nuclear protein | Cellular Transport | PAAG_04651 | YLR293C  GTP-binding nuclear protein |
| Calnexin | Protein fate (folding, modification, destination) | PAAG_07037 | YAL058W  Calnexin |
| Ubiquitin^+^ | Protein fate (folding, modification, destination) | PAAG_07080 | YLL039C  Ubiquitin |
| 26S proteasome regulatory subunit 3 | Protein fate (folding, modification, destination) | PAAG_05962 | YER021W  26S proteasome regulatory subunit 3 |
| Autophagy-related protein3 | Protein fate (folding, modification, destination) | PAAG_00493 | YNR007C  Protein autofagocitose |
| Atp-dependent RNA helicase | Protein synthesis | PAAG_05480 | YLL008W  Atp-dependent RNA helicase |
| Translation initiation factor 2-α kinase | Protein synthesis | PAAG_0659 | YJR007W  Translation initiation factor 2-α kinase |
| Sulfite oxidase | Nucleotide Metabolism | PAAG_07811 | YBR213W  Protein biosynthesis siroheme |
| Serine/threonine kinase | Signal transduction | PAAG_06510 | YBL105C  Serine/threonine kinase |
| Arrestin | Cell Cycle and DNA Processing | PAAG_03520 | YGL045W  pH-response regulator protein |
| Hypothetical protein* | --- | PAAG_06451 | YMR151W  Hypothetical protein |
| Hypothetical protein* | --- | PAAG_04310 | YKR031C  Phospholipase D1 |

^a^Function defined from the PEDANT 3 database[18] and MIPS [16].

^b^Accession number (PAAG) refer to structural genome databases of *Paracoccidioides* [54].

^c^Data obtained from *Saccharomyces* Genome Database – SGD [53].

*identity was not found in the databases used.

^+^Interactions confirmed by *in silico* experiments.
