# Supplementary material for: Intermolecular interactions of the malate synthase of Paracoccidioides spp
Source: BMC Microbiol. 2013 May 14;13:107. doi: 10.1186/1471-2180-13-107 (PMC3771410; doi:10.1186/1471-2180-13-107)
Supplement: Additional file 5: Table S4 — PbMLS-interacting proteins already described in the database interactions The GRID indicated in Figure 1. [file 1471-2180-13-107-S5.docx]

**Additional file 5: Table S4 - *Pb*MLS-interacting proteins already described in the database interactions The GRID indicated in Figure 1.**

| **Protein**  ***Paracoccidioides*** | **Protein**  ***S. cerevisiae*** |
| --- | --- |
|  |  |
| Ubiquitin  (PAAG_07080) | Ubiquitin  (YLL039C) |
| DNA repair protein  (PAAG_07058) | DNA repair protein  (YNL250W) |
| cAMP dependent protein kinase  (PAAG_00108) | cAMP dependent protein kinase  (YKL166C) |
| Peroxin  (PAAG_08209) | Peroxisomal membrane protein  (YOL147C) |
| Pyruvate carboxylase  (PAAG_00726) | Pyruvate carboxylase  (YGL062W) |
| Pyruvate carboxylase  (PAAG_00726) | Pyruvate carboxylase  (YBR218C) |
| Serine threonine kinase  (PAAG_06726) | Serine threonine kinase  (YGL158W) |
| Hypothetical protein  (PAAG_04321) | Hypothetical protein  (YGL059W) |

Data were obtained from the *Saccharomyces* Genome Database – SGD [53] and structural genome database of *Paracoccidioides* and *S. cerevisiae* [54, 23].
