# Supplementary material for: Intermolecular interactions of the malate synthase of Paracoccidioides spp
Source: BMC Microbiol. 2013 May 14;13:107. doi: 10.1186/1471-2180-13-107 (PMC3771410; doi:10.1186/1471-2180-13-107)
Supplement: Additional file 6: Table S5 — 3D Models informations of PbMLS and PbMLS-interacting proteins. [file 1471-2180-13-107-S6.doc]

**Additional file 6: Table S5 - 3D Models informations of *Pb*MLS and *Pb*MLS-interacting proteins.**

| **Proteins** | **Template** | **Template** | **Quality** |  | **RMSD^3^** | **RMSD^4^** | **Secondary** | | **Structure** | |  |
| --- | --- | --- | --- | --- | --- | --- | --- | --- | --- | --- | --- |
|  |  |  |  |  |  |  | **Modeller** | | **MD** | |  |
|  |  |  |  |  |  |  |  |  |  |  |  |
|  |  |  |  |  |  |  |  |  |  |  |  |
|  |  |  | **factor of** | **Time** | **template** |  |  |  |  |  |  |
|  |  |  |  |  |  |  |  |  |  |  |  |
|  | **PDB code** | **identity** |  |  |  | **Modeller** |  |  |  |  |  |
|  |  |  | **Modeller-** | **MD^2^** | **and** |  | **Helix** | **Sheet** | **Helix** | **Sheet** |  |
|  | **(resolution)** | **(coverage)** | **Model^1^** |  | **Modeller** | **and DM** |  |  |  |  |  |
|  |  |  |  |  |  |  |  |  |  |  |  |
|  |  |  |  |  |  |  |  |  |  |  |  |
| Enolase | 2AL1  (1.5Å) | 61% (98%) | 82.785 | 20 ns | 2.65Å | 3.13Å | 44.9% | 14.6% | 40.4% | 15.1% |  |
|  |  |  |  |  |  |  |  |  |  |  |  |
| Fructose 1,6 bisphosphate aldolase | 3QM3 (1.85Å) | 54% (98%) | 81.429 | 20 ns | 1.44Å | 3.97Å | 50.4% | 11.1% | 45.1% | 11.4% |  |
| Gamma actin | 1D4X (1.75Å) | 84% (99%) | 89.560 | 30 ns | 0.32Å | 2.82Å | 44.4% | 19.9% | 41.4% | 19.6% |  |
| Glyceraldehyde-3-phosphate dehydrogenase | 1DSS  (1.88Å) | 69% (99%) | 86.196 | 20 ns | 0.33Å | 3.16Å | 32.0% | 24.6% | 25.7% | 23.4% |  |
| Malate dehydrogenase | 2DFD  (1.9Å) | 56% (91%) | 95.455 | 20 ns | 0.48Å | 3.03Å | 49.4% | 18.4% | 41.8% | 17.4% |  |
|  |  |  |  |  |  |  |  |  |  |  |  |
| 2-Methylcitrate synthase | 3ENJ  (1.78Å) | 51% (98%) | 84.382 | 40 ns | 0.67Å | 6.34Å | 62.3% | 3.4% | 55.4% | 3.4% |  |
|  |  |  |  |  |  |  |  |  |  |  |  |
| Triosephosphate isomerase | 2I9E  (2Å) | 57% (94%) | 87.395 | 30 ns | 0.23Å | 2.87Å | 47.8% | 15.4% | 42.9% | 15.4% |  |
|  |  |  |  |  |  |  |  |  |  |  |  |
| Ubiquitin | 3B08  (1.7Å) | 96% (100%) | 89.362 | 60 ns | 0.91Å | 7.65Å | 25.2% | 33.1% | 14.6% | 32.5% |  |
| Malate synthase | 3CUZ (1.04Å) | 49% (97%) | 76.209 | 20 ns | 0.77Å | 2.55Å | 50.9% | 12.3% | 48.4% | 13.1% |  |
|  |  |  |  |  |  |  |  |  |  |  |  |

^1^Value obtained by ERRAT web server.

^2^Total time required to achieve stability RMSD in molecular dynamics simulations.

^3^RMSD between template and modeller structures superposed with respect to the backbone atoms.

^4^RMSD between modeller and MD structures superposed with respect to the backbone atoms.
