# Supplementary material for: Intermolecular interactions of the malate synthase of Paracoccidioides spp
Source: BMC Microbiol. 2013 May 14;13:107. doi: 10.1186/1471-2180-13-107 (PMC3771410; doi:10.1186/1471-2180-13-107)
Supplement: Additional file 7: Table S6 — Key residues and scores of the protein-protein interaction interface. [file 1471-2180-13-107-S7.docx]

**Additional file 7: Table S6 - Key residues and scores of the protein-protein interaction interface.**

| ***Pb*MLS-interacting proteins** | **Chain^1^** | **Key Residues** | **Global Energy^2^** |
| --- | --- | --- | --- |
| Enolase | A | ARG190; SER200; THR202; GLU203; GLU347; ARG370; SER371; ASN378 | -6.65 |
|  | B | ASP86; ASP107; ARG153; ASP155; LYS372; THR375; **ASP379**; **GLN380**; THR384 |  |
|  |  |  |  |
| Fructose 1,6 bisphophate aldolase | A | ARG302; LEU312; SER314; ALA315; LYS327; ARG333 | -143.74 |
|  | B | ALA74; PRO237; GLY239; ARG242; **ASP265**; PRO385; ARG391 |  |
| Gamma actin | A | ARG40; GLN42; THR67; GLU73; HIS74; ASN79; SER266; GLU271 | -21.31 |
|  | B | **LYS76**; ALA81; **GLY83**; ASP232; TYR233; ARG238; ARG264; **ASN386** |  |
| Glyceraldehyde-3-phosphate dehydrogenase | A | TYR41; TYR44; TYR48; GLN204; ASN205; ILE206 | -55.16 |
|  | B | ASP73; LYS76; ARG139; LYS157; LEU161; GLY206; **LEU388** |  |
|  |  |  |  |
| Malate dehydrogenase | A | ASP111; ASP112; THR187; ARG228; ASP241 | -65.54 |
|  | B | ASP298; LYS339; **ASP401**; ASN440 |  |
| 2-Methylcitrate synthase | A | SER68; THR69; ASP162; HIS164; LEU304; GLU311; LYS341; SER342; GLN344; VAL345; LYS399 | -67.35 |
|  | B | SER297; HIS396; ASN400; **ASP401**; ASN404; ASN406; ARG410; LYS418; ASN419 |  |
| Triosephosphate isomerase | A | THR16; ALA17; LEU100; ARG102 | -79.55 |
|  | B | VAL201; ALA205; ARG320; **LEU388** |  |
| Ubiquitin | A | LYS82; THR88; LYS158; THR161; ARG194; THR218 | -103.45 |
|  | B | **GLY83; ASP265; ASP379; GLN380; ASN386;** PHE389 |  |
|  |  |  |  |

^1^Chain-A refers to the *Pb*MLS-interacting proteins and chain-B to *Pb*MLS (receptor).

^2^Global energy according Fiberdock involved in the interaction between *Pb*MLS and *Pb*MLS-interacting proteins. Residues in bold are those from *Pb*MLS interface in contact with more than one protein.
